# Testing different models of pharmacy-based HIV pre- and post-exposure prophylaxis initiation and management in Kenya: protocol for a cluster-randomized controlled trial

**DOI:** 10.1186/s13063-025-09384-7

**Published:** 2025-12-30

**Authors:** Tabitha Kareithi, Stephanie D. Roche, Allison Meisner, Victor Omollo, Patricia A. Ong’wen, Kendall Harkey, Catherine Kiptinness, Peris Otieno, Lawrence Juma, Rachel C. Malen, Micah O. Anyona, Kelly Curran, Preetika Banerjee, Eunice Gichuru, Magdaline Asewe, Kaiyue Yu, Jillian Pintye, Melissa L. Mugambi, Parth D. Shah, Monisha Sharma, Daniel Were, Kenneth Ngure, Elizabeth A. Bukusi, Katrina F. Ortblad, Pharm PrEP cRCT team

**Affiliations:** 1 Partners in Health Research and Development, Center for Clinical Research, Kenya Medical Research Institute, Nairobi, Kenya; 2 Public Health Sciences Division, Fred Hutchinson Cancer Center, Seattle, WA USA; 3 Centre for Microbiology Research, Kenya Medical Research Institute, Kisumu, Kenya; 4 Jhpiego Kenya, Nairobi, Kenya; 5 Department of Global Health, University of Washington, Seattle, WA USA; 6 School of Public Health, Jomo Kenyatta University of Agriculture and Technology, Nairobi, Kenya

**Keywords:** Pharmacy, Pre-exposure prophylaxis (PrEP), Post-exposure prophylaxis (PEP), HIV prevention, Differentiated service delivery (DSD), Implementation science, Kenya

## Abstract

**Background:**

In Kenya, as in many African countries, private pharmacies are ubiquitous, frequently accessed, and underutilized for the delivery of HIV prevention services. Whether enabling private pharmacies to initiate and manage clients on HIV pre- and post-exposure prophylaxis (PrEP and PEP) leads to greater uptake and continuation than the current standard—pharmacy referral to clinic-based PrEP/PEP—is unknown. To address this gap and inform how private pharmacies might partner with the public sector, we are testing several models of pharmacy-delivered PrEP/PEP in comparison to the current standard.

**Methods:**

The Pharm PrEP cRCT is a 60-pharmacy, four-arm cluster-randomized controlled trial ongoing in Central and Western Kenya (first enrollment: 26 June 2023). Eligible pharmacies were licensed by the government, had a private room, and were willing to complete research activities (including a 3-day provider training). Study pharmacies were randomized 1:1:1:1 to (1) *client-sustained delivery*, in which clients pay pharmacies 250 KES (~$2 USD) per PrEP/PEP visit; (2) *implementor-sustained delivery*, in which clients pay nothing and implementors pay pharmacies 250 KES per PrEP/PEP visit; (3) *implementor-sustained* + *counselor-supported delivery*, in which clients pay nothing, delivery is supported by an HIV testing services (HTS) counselor, and implementors pay pharmacies 100 KES (~$1 USD) per PrEP/PEP visit; or 4) *referral (control),* in which clients pay nothing and implementors pay pharmacies 100 KES per referral to clinic-based PrEP/PEP. Pharmacies delivering PrEP/PEP receive supporting commodities free from government stock. Primary outcomes are PrEP initiation and continuation (any refilling) reported by clients 60 days post-enrollment; secondary outcomes include PEP initiation, PEP-to-PrEP transition, repeat PEP use, PrEP/PEP initiation, and PrEP/PEP continuation at 60 and 270 days post-enrollment. Primary analyses will compare each intervention arm to the control; secondary analyses will compare intervention arms to one another. We will additionally assess implementation outcomes (e.g., acceptability, feasibility, cost) from client and provider perspectives.

**Discussion:**

This trial will generate evidence on the potential benefits of leveraging private pharmacies for delivery of PrEP and PEP and the relative effectiveness of pharmacy delivery when subsidized by clients, implementors, and/or supported by HTS counselors. The findings may inform enabling policy and approaches for scale-up.

**Trial registration:**

ClinicalTrials.gov NCT05842122. Registered on April 5, 2023.

**Supplementary Information:**

The online version contains supplementary material available at 10.1186/s13063-025-09384-7.

## Introduction

### Background and rationale

In 2024, there were 450,000 new HIV infections in Eastern and Southern Africa [1], 34% of all global infections; to help curb the HIV epidemic, delivery models that increase access to highly effective biomedical HIV pre- and post-exposure prophylaxis (PrEP and PEP) are needed [2–4]. In many African countries, oral PrEP and PEP services are primarily delivered for free at public healthcare clinics, where client barriers to access—including long travel distances and wait times, lack of privacy, and HIV stigma—challenge initiation and continuation [5, 6]. Additionally, although the national guidelines of many African countries recommend PEP use for all individuals with recent HIV exposure [7], PEP continues to be offered primarily to individuals reporting occupational HIV exposure or sexual assault [8, 9]; provider bias and low public awareness of PEP further contribute to its underutilziation [10, 11]. New models of PrEP/PEP delivery outside of traditional clinic settings could potentially reach additional individuals who could benefit.

In Kenya, daily oral PrEP has been available in public healthcare clinics since 2017 and PEP since 2001. National guidelines specify that daily oral PrEP—tenofovir disoproxil fumarate with emtricitabine or lamivudine (TDF+FTC/3TC)—should be offered to individuals ≥ 15 years old who are not living with HIV (confirmed through rapid antibody testing), do not have medical contraindications (e.g., no renal or liver disease), and are at substantial ongoing HIV risk (with assessment guided by the Ministry of Health’s [MOH’s] 12-item HIV risk assessment screening tool [RAST]) [7]. For PEP, the guidelines recommend 28 days of tenofovir disoproxil fumarate/lamivudine or emtricitabine/dolutegravir (TDF + FTC/3TC + DTG) to individuals who are not living with HIV (confirmed through rapid antibody testing) and experienced, in the past 72 h, an exposure associated with high HIV acquisition risk (e.g., exposure to blood or semen of an individual potentially living with HIV). The guidelines also recommend creatinine, hepatitis B, and hepatitis C testing for PrEP and PEP initiation but note that initiation can proceed if these tests are unavailable (as is common). In 2021, Kenya approved the monthly dapivirine PrEP vaginal ring and, in 2024, the twice-monthly long-acting cabotegravir (CAB-LA) PrEP injections; however, few public healthcare clinics currently offer these products due to supply shortages. Overall, the guidelines recommend engaging individuals in biomedical HIV prevention services during periods of HIV acquisition risk, which may include continued PrEP or repeat PEP use based on client preferences, though individuals with repeat PEP use are encouraged to initiate PrEP.

To expand access to oral PrEP and PEP, Kenya and several other African countries are interested in leveraging private, community pharmacies, which are ubiquitous in many areas and a common first stop for sexual and reproductive health products, such as condoms, contraception, and treatment for sexually transmitted infections. In formative qualitative research led by our team, many pharmacy clients in Kenya cited convenience, privacy, and quality of care as reasons why they prefer accessing health services at private pharmacies over traditional healthcare clinics [12–15]. In two pilot studies led by our team, we demonstrated the feasibility of a pharmacy PrEP/PEP delivery model in Kenya in which trained providers—whose usual scope of practice does not include prescribing or HIV rapid diagnostic testing (RDT)—utilize a prescribing checklist to initiate and manage clients on PrEP and/or PEP under remote clinician oversight [6, 15, 16]. In these pilots, we also demonstrated that pharmacy PrEP/PEP delivery is in demand, can reach individuals underrepresented in clinic-based PrEP/PEP programs (e.g., unmarried individuals), and can achieve levels of PrEP continuation comparable with those observed at public clinics [15, 16]. Key challenges to model scale-up, however, include policy and regulatory gaps [17], uncertainty regarding sustainable financing mechanisms, and known barriers to implementation feasibility, such as low staffing ratios (typically one pharmacy provider per shift) and PrEP/PEP delivery time burden (~40 min per visit, with most of this time spent delivering counseling and HIV testing services).

### Objectives and trial design

To generate evidence to inform whether and how Kenya implements PrEP/PEP services at private pharmacies, we are conducting a superiority, cluster-randomized controlled trial (cRCT) comparing three pharmacy-delivered PrEP/PEP models to a “control” model that represents what pharmacy providers are currently legally allowed to do in Kenya: screen and refer potential PrEP/PEP candidates to clinic-based services. We hypothesize that enabling pharmacy providers to initiate and manage clients on PrEP and PEP at private pharmacies will lead to higher rates of PrEP initiation and continuation compared to pharmacy referral to clinic-based services.

**Fig. 1:**
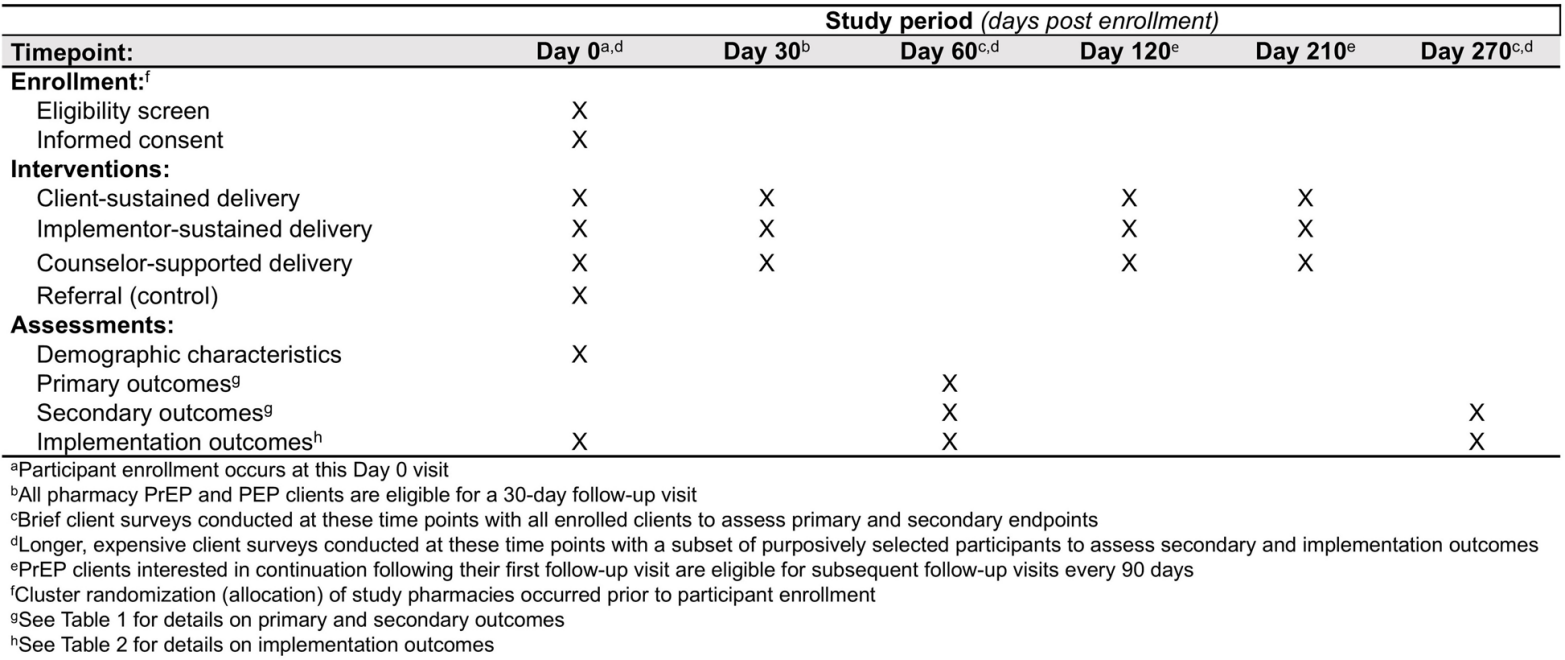
SPIRIT reporting guidelines for interventional trials

We report our cRCT methodology in accordance with the Standard Protocol Items: Recommendations for Interventional Trials guidelines [18] (Fig. 1, Additional File 1).

## Setting and participants

### Study setting

**Fig. 2:**
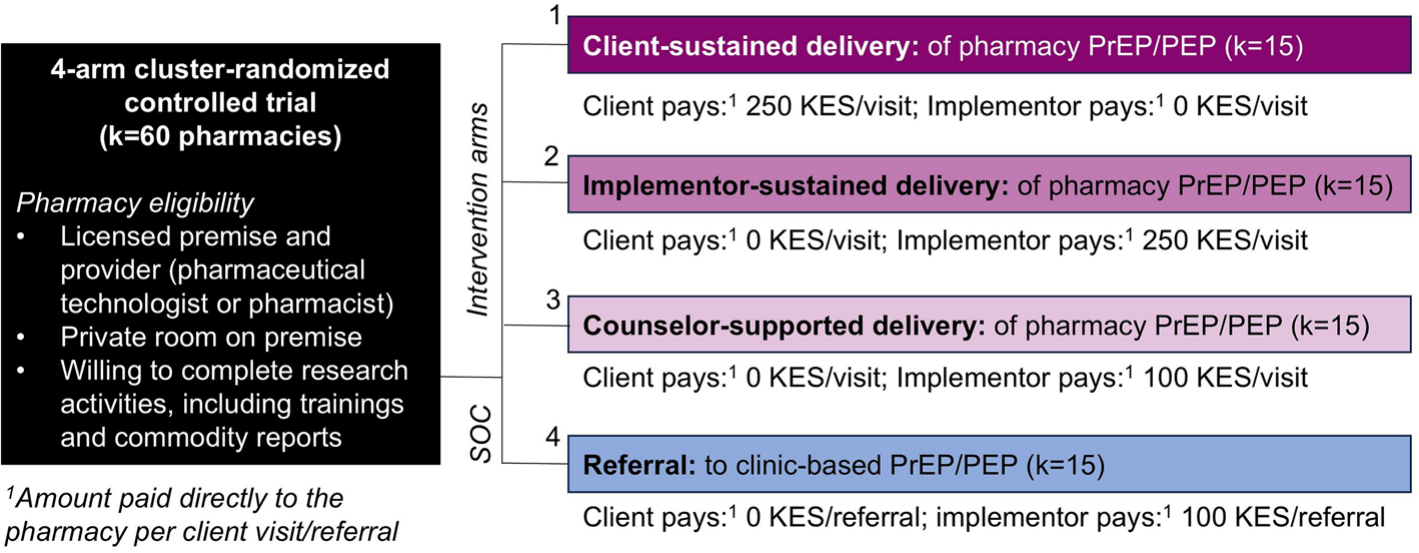
Study design for the Pharm PrEP cRCT

**Fig. 3:**
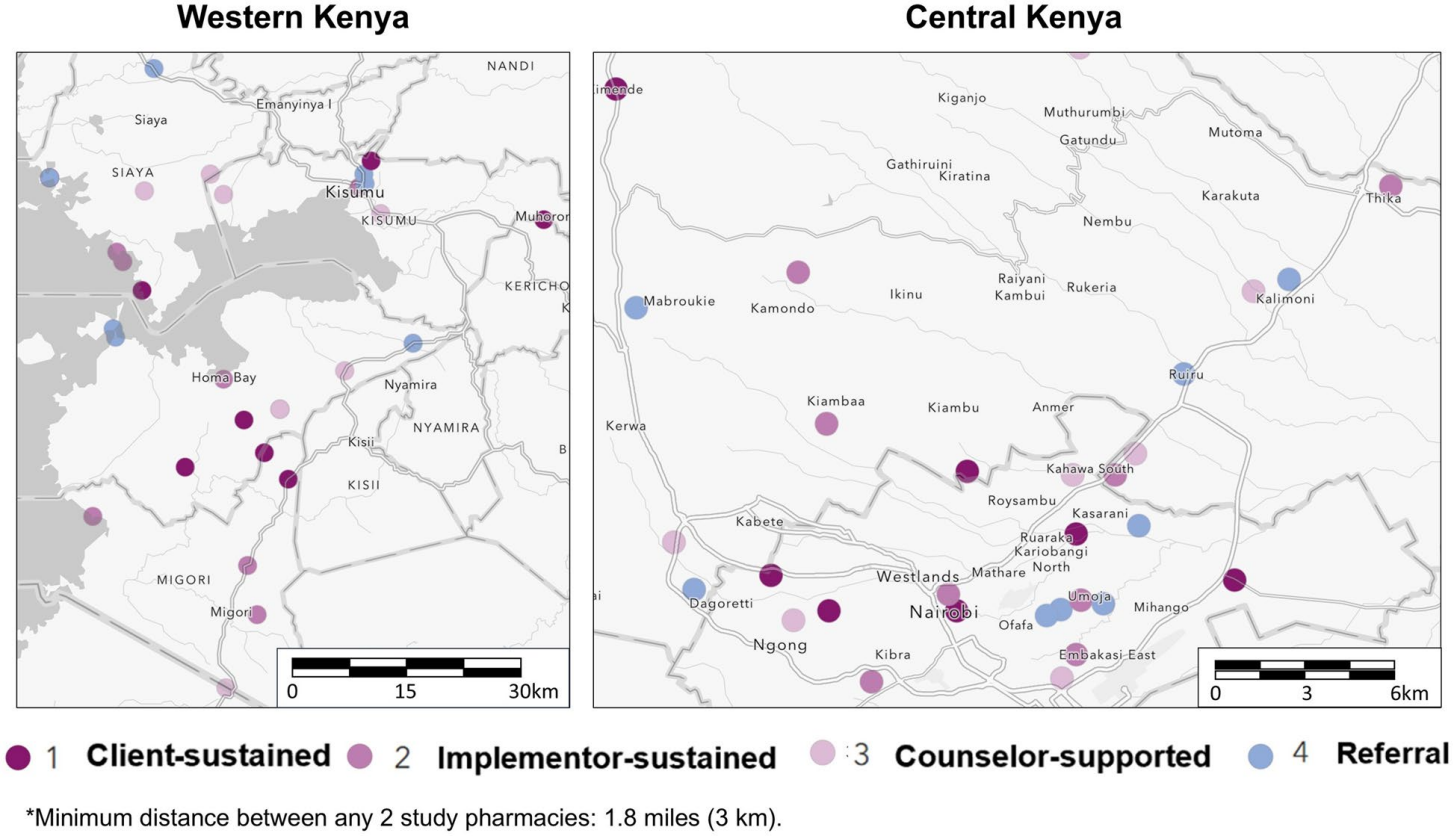
Location of pharmacies randomized to the different study arms

Our four-arm cRCT—the “Pharm PrEP cRCT”—includes 60 community pharmacies or “clusters” (k), equally distributed across 4 county groups (6 counties total) in Central and Western Kenya (ClinicalTrials.gov: NCT05842122) (Figs. 2 & 3). In Central Kenya, we are operating in Nairobi County (*k* = 15) and Kiambu County (*k*= 15), which include a mix of urban and peri-urban areas surrounded by rural areas and that have a population-level HIV prevalence of ~2–4% [19]. In Western Kenya, we are operating in Kisumu and Siaya counties (*k* = 15, treated as one county group) and in Homa Bay and Migori counties (*k*= 15, also treated as one county group); collectively, these two county groups include urban centers surrounded by large rural fishing and farming areas and have a population-level HIV prevalence of ~10–12% [19].

### Pharmacy eligibility, recruitment, and selection

Pharmacies were eligible for cRCT participation if they were registered with Kenya’s Pharmacy and Poisons Board, were willing to participate in study activities, and had the following: a current practicing license, a full-time licensed pharmacist or pharmaceutical technologist, and a private room for HIV testing and counseling. We collaborated with county and sub-county health management teams to identify eligible pharmacies located near hotspots for sexual activity, such as universities, bars, and trucking routes. To reduce the risk of contamination between study arms, we ensured at least 3 km of distance between study pharmacies. In each county group, we identified 20 pharmacies: 15 for participation and 5 “backups” in the event of study pharmacy attrition.

### Clinic recruitment and engagement

In each county, we worked with the county and sub-county health management teams to identify public, private, and faith-based healthcare clinics located near study pharmacies to which clients could be referred for PrEP, PEP, antiretroviral treatment (ART), and other services, as needed. Additionally, we identified public healthcare clinics (hereafter, “linked facilities”) from which study pharmacies will procure PrEP, PEP, and HIV testing kits; in return, study pharmacies complete monthly dispensing reports for their linked facility, which will, in turn, incorporate the pharmacy data with their own, submitting a final report—along with resupply requests—to the Kenya MOH.

### Pharmacy provider training

All pharmacy providers completed a three-part training: (1) A *self-paced online course*, covering HIV RDT, oral PrEP, and PEP; (2) a *2-day interactive in-person course*, to reinforce the online content, review study procedures and logistics, and practice HIV testing (this was implemented by county group); and (3) an *observed practice of HIV testing*, overseen by a Kenya MOH HTS supervisor. In collaboration with the Kenya MOH, we adapted and abbreviated the national PrEP and HIV testing curricula [20, 21] for pharmacy providers, focusing on the skills and competencies needed for PrEP/PEP delivery that are not part of standard pharmacist or pharmaceutical technologist training programs.

### Participant eligibility and recruitment

Eligible pharmacy clients are ≥ 16 years old, are interested in PrEP or PEP, meet all criteria on the prescribing checklist (described below), and are willing to consent to study activities. Eligible pharmacy providers, HTS counselors, and remote clinicians are ≥ 18 years old and involved in the implementation of pharmacy PrEP/PEP delivery or referral. Pharmacy client recruitment is performed across study arms by the study-trained pharmacy providers who are encouraged to display study-supplied posters about PrEP and PEP in their pharmacies (Additional Files 2 & 3); ask clients seeking sexual and reproductive health products (e.g., condoms, emergency contraception) if they are interested in, or in need of, PrEP or PEP; and share MOH PrEP informational brochures with potentially interested clients.

### Ethics and consent

The Scientific Ethics Review Unit of the Kenya Medical Research Center (KEMRI, Nairobi, Kenya) and Institutional Review Board (IRB) of the Fred Hutchinson Cancer Center (Fred Hutch, Seattle, United States [US]) approved this study protocol (Additional Files 4, 5, 6). Pharmacy clients complete verbal informed consent after being determined preliminarily eligible for PrEP/PEP and prior to HIV testing or referral; we received a waiver of parental consent for clients < 18 years old. Pharmacy providers, HTS counselors, and remote clinicians complete written informed consent prior to engagement in surveys or interviews. Study participants receive 250–500 Kenyan shillings (KES; ~$2–4 US dollars [USD]) for completing each research activity (e.g., survey), with the exact amount dependent on data collection tool length. Beyond the fee-for-service amount specified by their study arm assignment (detailed in the next section), the only additional compensation study pharmacies receive is ~5000 KES (~$40 USD) for completing a pharmacy volume tracking activity (conducted three times).

## Interventions

Study pharmacies were randomized 1:1:1:1 to one of four arms: (1) *Client-sustained delivery*, in which clients pay 250 KES (~$2 USD) per pharmacy PrEP/PEP visit; (2) *implementor-sustained delivery*, in which clients pay nothing and implementors pay 250 KES per PrEP/PEP visit; (3) *implementor-sustained* + *counselor-supported delivery*, in which clients pay nothing, delivery is supported by an HTS counselor stationed at the pharmacy, and implementors pay 100 KES (~$1 USD) per PrEP/PEP visit; or (4) *referral (control),*in which clients pay nothing and implementors pay 100 KES per PrEP/PEP referral. In line with Kenya’s interest in public–private partnerships for HIV service delivery [22], intervention arm pharmacies are supplied free PrEP/PEP drugs and HIV testing kits from government stock. Pharmacies are compensated for their time spent delivering PrEP/PEP via a fee-for-service model, with the payer and amount varying by arm. (Since pharmacy providers in the counselor-supported and referral arms are involved in fewer delivery activities, the compensation amount for these two arms is less.) [23] In the counselor-supported arm, we opted to provide pharmacies with an HTS counselor, rather than a higher cadre of healthcare provider (e.g., nurses), because HTS counselors are cheaper to employ, in greater supply, and competent in the most time-consuming PrEP/PEP delivery steps: the HIV risk assessment and HIV testing and counseling.

The Kenya-based teams implementing this cRCT include two KEMRI teams located in Kisumu and Kiambu counties and a team from Jhpiego Kenya, located in Nairobi County. These teams—which include site principal investigators (PIs), study coordinators, technical officers (TOs), research assistants (RAs), data coordinators, and laboratory technicians—have extensive experience conducting PrEP clinical and implementation research [2, 16, 24–26].

### Community involvement

We conducted extensive formative research [5, 6, 15, 16, 23, 27] and stakeholder consultation (with clients, providers, and county- and national-level policy makers) to determine the protocol for this trial, including the fees that would likely be affordable to a large segment of pharmacies’ clientele, motivating to pharmacy providers, and feasible for a public-sector payer to cover [23]. Additionally, the Jhpiego team is conducting extensive stakeholder engagement throughout the trial to address implementation challenges that may arise and advocate for complementary policies and guidelines that could enable and inform the scale-up of pharmacy-delivered PrEP/PEP services in Kenya.

### Procedures

**Fig. 4:**
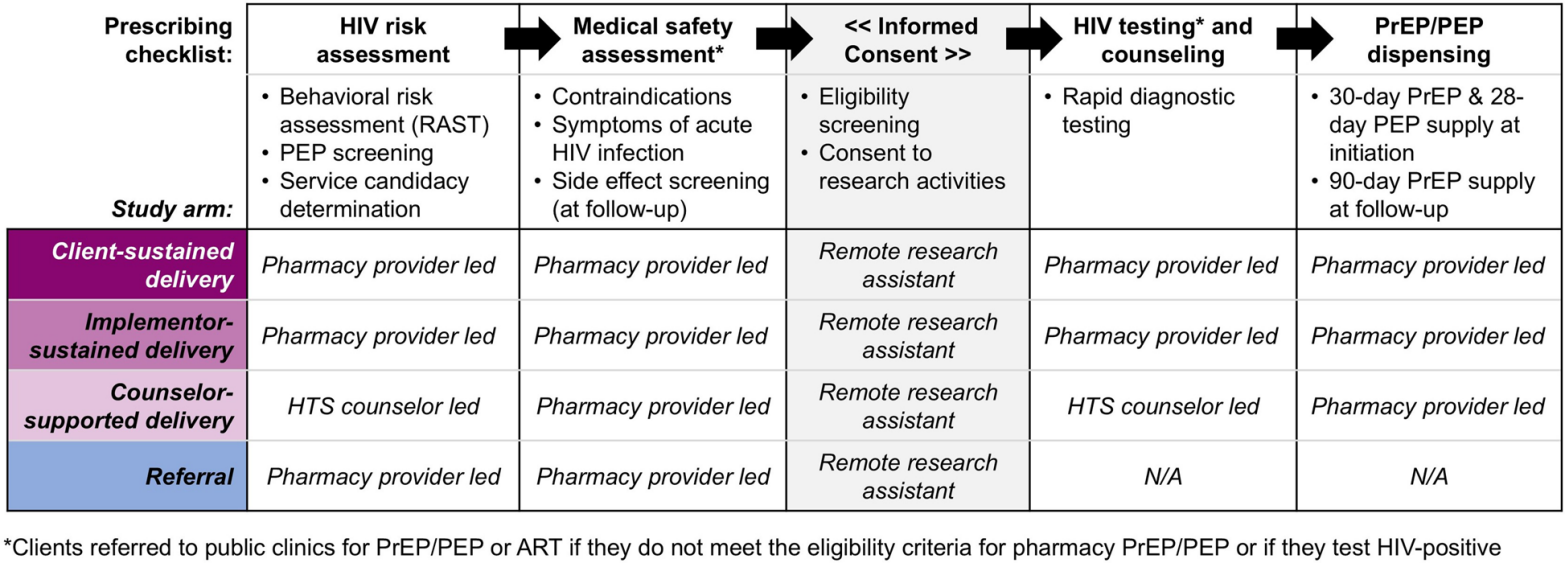
The core components of the prescribing checklist guiding pharmacy PrEP/PEP delivery or referral by study arm

In all arms, pharmacy providers deliver services using a standardized prescribing checklist (Fig. 4, see Additional File 7 for details). In collaboration with Kenyan policymakers [6], we developed, refined, and pilot tested [15] this checklist, which prompts providers to collect basic demographic details about the client and then guides them through the following core components of PrEP/PEP delivery: HIV risk assessment, medical safety assessment, HIV testing, and drug dispensing. (Providers in the referral arm use an abbreviated version of the checklist that includes only the client demographics section and HIV risk and medical safety assessments.) An electronic version of the checklist was built into a secure electronic point-of-sales system (Maisha Meds, Nairobi, Kenya) [28], with pharmacy providers able to complete the checklist on a portable tablet. The system also facilitates weekly payments to pharmacies not randomized to the client-sustained arm. A remote clinician provides oversight for PrEP/PEP prescribing and is available 24 h a day, 7 days a week via phone call or WhatsApp to address any pharmacy provider questions or concerns.

After a pharmacy client indicates interest in PrEP/PEP, the pharmacy provider invites them to a private room for eligibility assessment. HIV risk is assessed using a modified version of Kenya’s HIV RAST, which asks clients whether, in the past 6 months, they engaged in nine specific behaviors associated with HIV risk acquisition, such as engagement in transactional sex. We modified this tool to additionally ask clients if they perceive themselves at risk of HIV acquisition—regardless of their behaviors in the past six months, and if they had a potential HIV exposure—such as a condom break with someone who may be living with HIV or whose HIV status is unknown—in the past 72 h, which would qualify them for PEP. All clients are given the option to self-screen for HIV risk on the tablet, with the provider subsequently reviewing their responses and asking clarifying questions, as needed. Preliminary determination of PrEP or PEP candidacy is based on clients’ responses to RAST items and discussion with the pharmacy provider about their HIV prevention goals and preferences.

Next, pharmacy providers screen PrEP/PEP candidates for symptoms of acute HIV infection (e.g., fever, swollen lymph nodes, fatigue); PrEP candidates are additionally screened for a history of liver or kidney disease, diabetes, and hypertension. Across study arms, providers refer clients reporting any of these symptoms/conditions to nearby clinics for further evaluation. Clients who meet the eligibility criteria for pharmacy-based PrEP and PEP are invited to complete verbal informed consent over the phone with a study RA; during the consent, they all provide a phone number at which they can be reached for follow-up.

Following consent, clients at referral arm pharmacies receive information about nearby clinics delivering PrEP and PEP and are given a MOH-style referral form with information about their preferred referral clinic (e.g., location, hours). In intervention arm pharmacies, provider-administered HIV RDT and PrEP/PEP dispensing are delivered in accordance with national guidelines [7]. Clients who test HIV-positive are referred to nearby clinics for confirmatory testing and treatment, while clients who test HIV negative are eligible to receive either a 30-day supply of daily oral PrEP or a 28-day supply of daily oral PEP and are scheduled for follow-up 30 days later. To remind clients of follow-up visits, study pharmacies are encouraged to implement whatever systems (e.g., phone calls, SMS) they utilize for other products requiring follow-up (e.g., insulin). At PrEP follow-up visits, pharmacy providers again complete the prescribing checklist and additionally screen for potential PrEP side effects, dispensing those eligible to continue a 90-day PrEP supply. At PEP follow-up visits, pharmacy providers conduct follow-up HIV RDT, counsel on HIV risk reduction strategies, and offer PrEP eligibility screening.

To support delivery model implementation, trained TOs visit the pharmacies to provide routine technical assistance (TA) in service provision, commodity acquisition, and report completion. The frequency of TA visits varies based on how long the pharmacy has been participating in the study, the rate of participant enrollment, and the perceived level of support required.

### Outcomes

Our co-primary outcomes are PrEP initiation and continuation, assessed 60 days following enrollment (Table 1). We categorize PrEP initiation as being dispensed PrEP from any location—including at healthcare clinics—and PrEP continuation as refilling PrEP at any location following initiation (i.e., repeat dispensing). To ensure that these outcomes are assessed in a consistent manner across study arms (including for referral arm pharmacies that do not dispense PrEP/PEP and, as such, do not have dispensing records), we will use self-reported outcomes collected via client phone surveys for our primary analyses (described below). Survey data will also allow us to assess these outcomes among participants who obtain PrEP/PEP elsewhere (e.g., a private clinic) and never return to a study pharmacy for follow-up. We chose to assess this outcome 60 days following enrollment, as this would give control arm participants time to follow through on referrals to clinic-based PrEP/PEP services and all participants time to return for their first PrEP refill 30 days later.

**Table 1:** Primary and secondary outcomes of the Pharm PrEP cRCT

Outcomes	Definition	Timing	Data source
*Primary*			
PrEP initiation	Number (no.) of participants that initiated (i.e., were dispensed) PrEP at the pharmacy or clinic	60 days	Brief client surveys, clinical records*
PrEP continuation	No. of participants that initiated PrEP at the pharmacy or clinic, returned, and refilled PrEP	60 days	Brief client surveys, clinical records*
*Secondary*			
PrEP initiation	No. of participants that initiated (i.e., were dispensed) PrEP at a pharmacy or clinic	270 days	Brief client surveys, clinical records*
PrEP continuation	No. of participants that initiated PrEP at a pharmacy or clinic, returned, and refilled PrEP No. of participants that initiated PrEP at a pharmacy or clinic and *refilled PrEP at least twice*	270 days	Brief client surveys, clinical records*
PrEP re-initiation	Number of participants that initiated PrEP at a pharmacy or clinic and *refilled PrEP > 7 days after their expected refill date* (i.e., stopping and restarting)	270 days	Brief client surveys, clinical records*
PrEP adherence	No. of participants who initiated PrEP at the pharmacy or clinic and self-reported behaviors indicative of good adherence^a^	60 days	Brief client surveys, clinical records*
PrEP-to-PEP transition	No. of participants that successfully completed PEP and were found eligible for and initiated PrEP at the pharmacy or clinic	60, 270 days	Brief client surveys, clinical records*
PEP initiation	No. of participants that initiated (i.e., were dispensed) PEP at the pharmacy or clinic within 72 h of screening	60, 270 days	Brief client surveys, clinical records*
PEP-to-PrEP transition	No. of participants that successfully completed PEP and were found eligible for and initiated PrEP at the pharmacy or clinic	60, 270 days	Brief client surveys, clinical records*
Recurrent PEP use	No. of participants that were dispensed PEP more than one time	60, 270 days	Brief client surveys, clinical records*
PEP/PrEP initiation	No. of participants that initiated (i.e., were dispensed) either PEP or PrEP at the pharmacy or clinic	60, 270 days	Brief client surveys, clinical records*
PEP/PrEP continuation	Number of participants that (1) initiated PrEP and refilled PrEP or later were dispensed PEP or (2) initiated PEP and transitioned to PrEP or later were dispensed PEP again	60, 270 days	Brief client surveys, clinical records*
*Tertiary*			
Follow-up HIV testing	Number of participants engaging in HIV testing following the pharmacy PrEP/PEP visit	60, 270 days	Brief client surveys, clinical records*
Behaviors associated with HIV risk	Any and specific self-reported behaviors associated with HIV risk acquisition according to Kenya’s Rapid Assessment Screening Tool	60, 270 days	Brief client surveys, clinical records*
PrEP coverage	Duration of time on PrEP, based on dates of dispensing and the quantity of pills dispensed at each visit using all longitudinal data available at completion of the cRCT	Study duration	Brief client surveys, clinical records*

*The clinic records will be abstracted from participating study pharmacies and nearby public healthcare clinics to which clients were referred. These data will be used for secondary analyses in which we compare the intervention arms to one another (using pharmacy record data)

^a^Using the Wilson et al*. *scale; 2020, *AIDS*

Secondary outcomes assessed 270 days following enrollment include PrEP initiation, PrEP continuation (including any and ≥ 2 refills), and PrEP re-initiation (> 7 days after a scheduled follow-up visit). We chose to measure these outcomes 270 days (~9 months) following enrollment so that PrEP clients would have sufficient time to refill PrEP at least three times. Secondary outcomes assessed 60 days and 270 days following enrollment include PrEP adherence (self-reported in client surveys), PrEP-to-PEP transition (i.e., any PEP dispensing following PrEP initiation), PEP initiation (i.e., any PEP dispensing), PEP-to-PrEP transition (i.e., any PrEP dispensing following PEP initiation), recurrent PEP use (i.e., repeat PEP dispensing), PrEP or PEP initiation, and PrEP or PEP continuation (i.e., dispensing of either product following initiation of PrEP or PEP).

All primary and secondary study outcomes are primarily analyzed as pharmacy-level count outcomes (described in detail below).

### Implementation outcomes

In addition to our primary and secondary outcomes, we will assess several implementation outcomes related to pharmacy PrEP/PEP delivery or referral among clients, providers, HTS counselors, and remote clinicians (with questions adjusted to participants’ study arm assignment) (Table 2). Among clients, we will assess perceptions of the acceptability [29] and quality [30] of PrEP/PEP services received and their willingness to pay for such services in the future; we will also ask questions about select parts of their pharmacy visit to assess fidelity to the delivery model (e.g., to see if HIV testing occurred in a private room). Among providers, we will assess perceptions of their assigned delivery model’s acceptability [29] and feasibility [31], confidence in their ability to deliver/refer (i.e., self-efficacy), and willingness to charge for PrEP/PEP service delivery/referral. At the pharmacy level, we will assess the sustainability and costs of the different pharmacy PrEP/PEP delivery models tested (i.e., study arms) and any model adaptations made [32] over the implementation period. To evaluate these outcomes, we will primarily use extensive client surveys and provider surveys, described elsewhere.

**Table 2:** Implementation outcomes assessed through the Pharm PrEP cRCT

Outcomes	Definition	Timing	Data source
*Client outcomes*			
Acceptability	Agreement (on 5-point Likert scale) to seven statements that measure different dimensions of acceptability posited by the Theoretical Framework of Acceptability^a^ (e.g., affective attitude, burden, perceived effectiveness)	Baseline, 60, 270 days	*Extensive client surveys*
Fidelity	Number (no.) and proportion of participants that received different core components of the intervention (e.g., counseling, HIV testing) as specified in the protocol	Baseline, 60, 270 days	*Extensive client surveys*
Willingness to pay	Amount pharmacy clients are willing to pay for each pharmacy PrEP or PEP visit	Baseline, 60, 270 days	*Extensive client surveys*
Quality of care perceptions	Agreement (on 7-point Likert scale) to six statements that measure different dimensions of service quality posited by the Perceived Service Quality Scale-Short Form (pSQ-SF6)^b^ (e.g., relationship quality, environmental quality), with higher scores (range: 6–42) indicating higher perceived service quality	Baseline, 60, 270 days	*Extensive client surveys*
*Provider outcomes*			
Acceptability	Agreement (on 5-point Likert scale) to five statements that measure different dimensions of acceptability posited by the Theoretical Framework of Acceptability^a^	Baseline, 60, 270 days	*Provider surveys*
Feasibility	Agreement (on 5-point Likert scale) to three statements comprising the Feasibility of Intervention Measure (FIM)^c^	Baseline, 60, 270 days	*Provider surveys*
Perceived self-efficacy	Agreement (on 5-point Likert scale) to 10 statements that assess providers’ level of confidence delivering different core components of the intervention	Baseline, 60, 270 days	*Provider surveys*
Willingness to charge	Amount pharmacy providers are willing to charge for each pharmacy PrEP or PEP visit under different scenarios	Baseline, 60, 270 days	*Provider surveys*
*Pharmacy outcomes*			
Sustainability	Comparison of primary and secondary outcomes during the first half and second half of implementation	Study period	*Brief client survey, clinic records*
Cost	Estimates of the total annual cost of program implementation and cost per outcome (e.g., PrEP initiation, PrEP continuation); model the cost-effectiveness (e.g., cost per DALY adverted) for each study arm	Study period	*Time-and-motion observations, pharmacy records*
Adaptations	Changes made to the delivery model, documented according to the modules of the Framework for Reporting Adaptations and Modifications to Evidence-based Implementation Strategies (FRAME-IS)^d^	Study period	*TA reports*

*Abbreviations*: *cRCT* cluster-randomized controlled trial, *DALY* disability-adjusted life year, *PEP* post-exposure prophylaxis, *PrEP* pre-exposure prophylaxis, *TA* technical assistance

^a^ Sekhon et al., 2017, *BMC Health Serv Res*

^b^ Carter et al., 2022, *Res Social Adm Pharm*

^c^ Weiner et al., 2017, *Implement Sci*

^d^ Miller et al., 2021, *Implement Sci*

### Power and sample size

We powered a four-arm 60-pharmacy trial based on our primary PrEP initiation and continuation outcomes, with primary comparisons between each intervention arm and the control arm. Originally, we planned to analyze differences in the proportion of enrolled clients seeking sexual and reproductive health services with these outcomes (i.e., an individual-level analysis comparing proportions across arms). However, in September 2023 (3 months into trial implementation), we switched to analyzing differences in the rate of pharmacy clients with these outcomes (i.e., a pharmacy-level analysis of counts adjusted for pharmacy volume, which can be conceptualized as a rate). This switch—supported by our Data Safety and Monitoring Board (DSMB)—was motivated by observed uneven enrollments across the study arms resulting from clients’ interest in the different services offered; if unaccounted for, the uneven enrollment would have biased the proportion of PrEP initiations and continuations in each arm.

To inform our sample size calculations, we used data on monthly PrEP and PEP initiations and continuation from two pilot studies, one that delivered PrEP services for a 300 KES (~$3 USD) client fee [16]—like the client-sustained arm—and another that delivered PrEP/PEP services for free [15]—like the implementor-sustained arm. Additionally, to inform assumptions on PrEP continuation in the referral arm, we used data from a large implementation project of clinic-delivered PrEP [26]. For all primary PrEP and select secondary PEP and PrEP/PEP outcomes (i.e., initiation and continuation), we hypothesized that the counselor-supported arm would perform best, followed by the implementor-sustained arm, client-sustained arm, and control arm. Additional File 8 summarizes the number of PrEP, PEP, and PrEP/PEP initiations and continuations we anticipated per pharmacy per month.

We estimated power for an analysis of cluster-level count data from a cRCT using the *clusterPower*package in R. In the setting of count outcomes, power is driven by differences in pharmacy-level rates (e.g., the number of PrEP initiations per pharmacy divided by the pharmacy volume) in each intervention arm compared to the control. To estimate power, we assumed a coefficient of variation of 0.25, typical for cRCTs [33], and a significance level of alpha = 0.05/3—which corresponds to a Bonferroni adjustment for multiple comparisons between the three intervention arms. Furthermore, we assumed that the average pharmacy volume (described in detail below) was the same across the four study arms; under this assumption, pharmacy volume does not influence our study power. Since power would be unaffected whether the pharmacy volume was 1 or 1000 in each arm; for simplicity, we chose a value of 1.

When our hypothesized monthly pace of enrollment was lower than anticipated in November 2024 (18 months into trial implementation), we estimated the number of PrEP initiations needed to detect the hypothesized differences in PrEP initiations between each intervention arm and the control with at least 80% power, assuming the same proportional relationships as in Additional File 8. Based on these calculations, 1119 total PrEP initiations across the study arms would give us 100% power to detect hypothesized differences in PrEP initiation between the implementor-sustained and counselor-supported arms vs. the referral arm and 80% power to detect the hypothesized difference between the client-sustained arm vs. the referral arm (Additional File 9). For our PrEP continuation outcome, this sample would also give us 100% power to detect hypothesized differences in the implementor-sustained and counselor-supported arms vs. the referral arm and 23% power to detect the hypothesized difference between the client-sustained arm vs. the referral arm—although large differences in PrEP continuation between these arms are not expected, this comparison has other important sustainability implications (i.e., client subsidization) for pharmacy-delivered PrEP and PEP services.

## Assignment of interventions

Study arm assignments were stratified by the four county groups and generated by the study biostatistician, author A. M., who was not involved in pharmacy selection (Additional File 10). Following the completion of the in-person provider training, one provider per study pharmacy randomly selected an opaque, sealed envelope indicating a study arm assignment or backup pharmacy designation. Due to the nature of the intervention, this trial is unblinded. Any time a study pharmacy drops trial participation for any reason (e.g., provider turnover, inability to follow study procedures), that pharmacy is replaced by a randomly selected backup within the same county group.

## Data collection and management

### Data collection

#### Survey data

All enrolled pharmacy clients are invited to complete brief surveys at 60 and 270 days post-enrollment. In these ~10-min phone-based surveys, trained RAs ask clients to self-report if they initiated PrEP, PEP, or ART (and where), engaged in and behaviors and perceptions related to HIV risk acquisition. For clients who initiated PrEP, we ask if they refilled or discontinued PrEP (and when); for clients who initiated PEP, we ask if they transitioned to PrEP and/or were dispensed PEP again (and when). Using Wilson et al.’s three-item adherence scale, [34] we ask clients who report PrEP use to self-report their adherence in the past month. Participants are considered unreachable after being contacted three times without response.

Additionally, a subset of clients (*n* = 1340) and all providers (*n* = 75, including HTS counselors) are invited to complete longer phone-based surveys at baseline, 60 days, and 270 days post-enrollment. In these surveys, we assess several of our proposed implementation outcomes (e.g., acceptability, feasibility, fidelity). We will use purposive sampling to collect a range of experiences from both PrEP (*n* = 670) and PEP (*n* = 670) clients across study arms. Whereas providers will be followed longitudinally, different subsets of clients will be surveyed at baseline (*n* = 540), 60 days (*n* = 400), and 270 days (*n* = 400).

#### Clinical records

Clinical records will be abstracted from participating study pharmacies and nearby referral clinics for use in secondary analyses (described below). Pharmacy data will come from the standardized electronic prescribing checklist used in all study pharmacies to inform PrEP/PEP dispensing or referral; referral clinic data will come from paper or electronic records. Both data sources include information on behaviors associated with HIV risk acquisition (from the Kenya RAST) and PrEP, PEP, and ART dispensing.

#### Pharmacy volume

Our primary and secondary analyses require estimates of study pharmacies’ monthly client volume. To estimate this, we will use a novel approach whereby we supply pharmacies with brown paper bags (typical packaging for items purchased at Kenyan pharmacies) and instruct pharmacy staff to use these bags (preferably one per client) for all clients, putting aside a bag if a client declines one for their items. We will then track the number of bags each pharmacy distributes weekly over the course of 1 month. To account for seasonal variation, we will conduct this activity at three different timepoints over the calendar year.

#### In-depth interviews (IDIs)

To contextualize our quantitative findings (e.g., identify factors that influenced client PrEP/PEP decision-making) and provide implementation insights—including barriers, facilitators, and recommended changes to each PrEP/PEP delivery/referral model—we will conduct IDIs with purposively sampled pharmacy clients (*n* = 148), providers and HTS counselors (*n* = 55), and remote clinicians (*n* = 4). For details on IDI sampling, see Additional File 11.

### Data management

All data from study eligibility screening, surveys, and abstracted clinic records is collected electronically and stored in Research Electronic Data Capture (REDCap) [35]. Pharmacy records are collected and stored in Maisha Meds. REDCap and Maisha Meds data are merged weekly using participants’ phone or identification numbers. In the case of discrepancies, data are checked against study sites’ internal records. As a quality control measure, both data entry platforms have error messages that alert users to out-of-range entries and/or ineligibility (e.g., for PrEP dispensing).

IDIs will be audio recorded with key points summarized by the interviewer in a debrief report and then transcribed verbatim (with non-English portions translated, as needed). All de-identified transcripts are saved in Microsoft Teams and uploaded to Dedoose (Sociocultural Research Consultants LLC, Manhattan Beach, USA) for analysis.

## Statistical methods

### Analysis of primary and secondary outcomes

Our primary analysis of PrEP initiation/continuation will be intention to treat and use data from all enrolled clients, including those enrolled in dropped study pharmacies. We will fit Poisson generalized linear models (GLMs) with log links and robust standard errors, where the outcome is pharmacy-level counts, the predictor of interest is study arm (modeled as a nominal or factor variable), and county group is adjusted to account for stratified randomization. Pharmacy volume (i.e., the monthly median from the three rounds of bag counting) is included as an offset term. Missing data will be imputed as failures. Given the offset and log link, this model can also be conceptualized as modeling an outcome rate (i.e., the number of PrEP initiations or continuations per pharmacy volume). For example, if a given pharmacy had 10 PrEP initiations in a month and their monthly volume was 500 clients, the PrEP initiation rate would be 1 per 50 clients. The log link in the Poisson GLM additionally means that the study arm coefficients reflect the log of the ratio of rates (i.e., the log of the rate ratio) between each intervention arm and the control. This is summarized for the primary outcomes in Table 3. Since this implementation study is considered minimal risk, there are no planned interim analyses or formal stopping rules.

**Table 3:** Summary of primary and secondary analyses of the primary outcomes

Outcome	Analysis	Model
PrEP initiation*	*Primary analysis*: Pharmacy level, where each pharmacy is summarized by total number of initiations (*count outcome*)	Poisson GLM with log link to estimate the ratio of PrEP initiation rates (no. of initiations per pharmacy volume) in each intervention arm vs. the control
PrEP continuation	*Primary analysis*: Pharmacy level, where each pharmacy is summarized by total number of continuations (*count outcome*)*	Poisson GLM with log link to estimate the ratio of PrEP continuation rates (no. of continuations per pharmacy volume) in each intervention arm vs. the control
	*Secondary analysis*: Individual level, where the analysis is limited to those who initiated and each person who initiated has an indicator for continuation (*clustered binary outcome*)	Poisson GEE with identity link and robust standard errors to estimate the risk difference (proportion continuing among those who initiated) in each intervention arm vs. the control. In this case, the *denominator* is the number who initiated in each arm

*Abbreviations*: *GEE* Poisson generalized estimating equations, *GLM* Poisson generalized linear models, *PEP* post-exposure prophylaxis, *PrEP* pre-exposure prophylaxis

* Powered analyses in the design of the study

### Sensitivity and secondary analyses

We will conduct several pre-specified sensitivity analyses, including one that accounts for gaps in implementation at each pharmacy and another that conducts a nonparametric permutation test to understand whether departures from the assumed statistical model affect the trial findings.

In secondary analyses, we will compare the primary outcomes in each intervention arm to one another (three unique comparisons) using data from pharmacy records as opposed to brief client surveys since the former is a more accurate data source that has PrEP/PEP dispensing information for all intervention arm participants. Additionally, we will test for differences in primary outcome intervention effects by urban/rural status and participant subgroups of interest (e.g., women, individuals < 25 years). Furthermore, we will compare the proportion of PrEP continuations among those who initiate PrEP in each of the intervention arms to the control arm (i.e., an individual-level analysis of a binary PrEP continuation indicator). Finally, we will compare PrEP initiation and continuation at 60 days when assessed using two different data sources: client self-report (in surveys) versus clinical records (i.e., PrEP/PEP dispensing documented by study pharmacies and referral clinics).

We will use the described Poisson GLM model for analyses of pharmacy-level count outcomes and a Poisson generalized estimating equations (GEE) model for analyses of individual-level binary outcomes (i.e., the proportion continuing PrEP among those who initiate). Since individual-level outcomes are clustered among individuals within the same pharmacy, methods for correlated data are required [36].

### Analyses of implementation outcomes and determinants

Most implementation outcomes will be reported descriptively from survey data. To estimate implementation costs in each intervention arm, we will conduct microcosting [37] using expense reports, provider surveys (that assess resource use and salaries), pharmacy records, and time-and-motion observations. IDI transcripts will be analyzed thematically by a team of US and Kenyan qualitative researchers using a combination of inductive and deductive approaches, the latter informed by the updated Consolidated Framework for Implementation Research [38] and Theoretical Domains Framework [39]. The qualitative researchers will meet on a routine basis throughout the analysis to discuss and build consensus around emerging themes.

## Oversight and monitoring

### Coordinating center

A team at the Fred Hutch serves as the coordinating center for the trial. This team arranges virtual weekly and as needed meetings with the Kenyan-based research and implementation teams (described above) to collaboratively manage the day-to-day trial operations, ensure trial protocols and survey tools are up to date, report noncompliance issues, perform data quality checks, and analyze trial data. Additionally, leaders from these teams—including the PIs, study coordinators/managers, and TOs—form a Project Management Group, which meets twice a month to review trial conduct and develop solutions to implementation challenges. All protocol noncompliance instances are documented and submitted to the overseeing IRBs within 10 days.

### DSMB

Trial implementation is being monitored by an independent DSMB, consisting of three researchers—two based in Kenya and one in the USA—with expertise in HIV prevention, implementation science, and biostatistics. The DSMB meets semiannually, with the research team presenting on study progress, implementation challenges and solutions, adverse events (e.g., severe drug side effects, social harms), and primary and secondary trial outcomes (aggregated across study arms in an open session and disaggregated by the study arms in a closed session). The DSMB makes recommendations related to trial implementation, participant safety, data management, quality control, and/or analysis. Detailed meeting minutes are shared with the overseeing IRBs.

### Protocol amendments

Any changes made to the protocol due to implementation challenges or DSMB suggestions will be investigator-initiated, submitted to the overseeing IRBs for approval, reported to the funder, and sent to the study sites for inclusion in their site documents. All relevant changes will also be updated in the Clinical Trials Registry.

### Confidentiality

Study sites will use standard operating procedures to ensure participants’ confidentiality before, during, and after trial implementation. Datasets containing participant information will be de-identified and stored in a separate location than records containing identifiable information (e.g., consent forms), which will also be securely stored.

## Dissemination plans

In line with principles of good participatory practice [40], we will disseminate study findings to diverse audiences in appropriate formats. To engage participants and other community members, we will present findings at community events and distribute flyers at study pharmacies. To reach Kenyan stakeholders, we will present findings and share policy briefs at national and regional meetings, as well as invited forums with government officials, regulators, researchers, and HIV program implementors. To reach global researchers and policymakers, we will publish peer-reviewed academic manuscripts and present our findings at international HIV conferences.

## Discussion

The three models of pharmacy-delivered PrEP and PEP being tested in the Pharm PrEP cRCT are designed to address different barriers that pharmacy clients and providers experienced in prior studies (e.g., cost for clients, time constraints for providers) and fill key evidence gaps on the potential benefits of pharmacy-based PrEP/PEP initiation and management compared to pharmacy referral to clinic-based PrEP/PEP services, which represents the existing standard if governments do not enact enabling policies. Additionally, this trial will generate important complementary evidence on intervention costs as well as barriers and facilitators to implementing the different delivery models from client and provider perspectives. With the inclusion of PEP, the study will also provide insights into clients’ needs and preferences for different biomedical HIV prevention products, generating much-needed evidence on the potential role PEP could play in HIV prevention programs.

Strengths of this study include the robust cRCT design, measurement of clinical and implementation outcomes using mixed methods, delivery of both PrEP and PEP products, three intervention arms testing different pharmacy PrEP/PEP delivery models, and availability of PrEP/PEP to all pharmacy clients, regardless of gender or age. Other strengths include the involvement of pharmacies across six Kenyan counties and two geographic regions, which will increase the generalizability of the study findings, and our partnership with Kenyan stakeholders, which will increase the likelihood that our findings influence the national scale-up of pharmacy-delivered PrEP/PEP services in Kenya.

This study also has limitations. First, our primary outcomes are assessed using self-reported survey data versus clinical records. However, we needed to assess these outcomes this way to ensure consistency in the data source used across study arms and to reach participants who never return to a study pharmacy. In secondary analyses, we will compare the self-reported outcomes with those observed in the clinical (pharmacy and clinic) records. Second, we were underpowered (< 80%) to detect differences in PrEP continuation between our client-sustained arm and referral arm but have > 80% power for all other primary comparisons of interest. Third, pharmacy-delivered PrEP/PEP services in the intervention arms are supported with government commodities, which lowers implementation costs compared to a non-supported delivery scenario. Finally, pharmacy providers in all arms receive TA from study staff to address implementation challenges, which may not be readily available in a scale-up scenario.

Evidence from this study could guide the Kenya MOH on how public PrEP/PEP commodities could be implemented and financed at private pharmacies in Kenya, which could increase the coverage and reach of biomedical HIV prevention products nationally. Though generated in Kenya, the trial insights could inform HIV programming in other countries pursuing private-sector engagement as a means to sustain and expand HIV service delivery—an approach that could help protect the hard-won advances against HIV despite sharp declines in foreign aid for HIV programs.

## Trial status

The first trial participant was enrolled on June 26, 2023; the anticipated last trial participant will be enrolled around July 2025. This trial was registered on ClinicalTrials.gov on April 5, 2023. All relevant ethics committees have approved the trial protocol and relevant modifications; the current protocol was version 2.2 at the time of this publication (December 2025).

## Supplementary Information

Additional file 1: SPIRIT Checklist as required by *Trials*.

Additional file 2: PrEP poster. This poster was printed out and hung up in all study pharmacies to advertise PrEP services

Additional file 3: PEP poster. This poster was printed out and hung up in all study pharmacies to advertise PEP services.
Additional file 4: Fred Hutch IRB approval. Ethical approval document—Fred Hutch IRB.
Additional file 5: SERU Kisumu site approval. Ethical approval document—SERU for Kisumu County, Kenya.
Additional file 6: SERU Kiambu site approval. Ethical approval document—SERU for Kiambu County, Kenya

Additional file 7: Prescribing Checklist. This prescribing checklist is what providers use to screen clients for PrEP/PEP eligibility. The checklist is uploaded onto a tablet for providers to complete

Additional file 8: Initiation assumptions. Number of PrEP and PEP initiations and continuations expected per pharmacy per month, by study arm. This table outlines the number of PrEP, PEP, and PrEP/PEP initiations and continuations we anticipated per pharmacy per month

Additional file 9: Power analysis for PrEP and PrEP/PEP outcome comparisons (alpha=0.5/3). This table outlines the estimated power calculations by arm and outcome, as well as the number of PrEP and PEP continuations needed by arm to meet >80% power

Additional file 10: Randomization scheme for the cRCT and back-up pharmacy distribution. This table outlines how many pharmacies were randomized by county and by arm

Additional file 11. Qualitative interviews_2025.11.05. This describes the methodological orientation for the qualitative research of this project, as well as the rationale for the type and number of in-depth interviews collected.
Additional file 12: Original funding document. BMGF INV-033052 investment document. The original funding document for this grant

## Data Availability

The study investigators will provide access to the trial protocol, data, and statistical code upon request.

## Abbreviations

AIDS: Acquired Immunodeficiency Syndrome
ART: Antiretroviral Treatment
CAB-LA: Cabotegravir
cRCT: Cluster-Randomized Controlled Trial
DSD: Differentiated Service Delivery
DSMB: Data Safety and Monitoring Board
DBS: Dried Blood Spot
FIM: Feasibility of Intervention Measure
FRAME-IS: Framework for Reporting Adaptations and Modifications to Evidence-based Implementation Strategies
GEE: Generalized Estimating Equations
GLM: Generalized Linear Models
HIV: Human Immunodeficiency Virus
HTS: HIV Testing Services
IDI: In-Depth Interview
IRB: Institutional Review Board
KEMRI: Kenya Medical Research Institute
KES: Kenya Shillings
MOH: Ministry of Health (Kenya)
PEP: Post Exposure Prophylaxis
PrEP: Pre-Exposure Prophylaxis
RAST: Risk Assessment Screening Tool
RDT: Rapid Diagnostic Testing
REDCap: Research Electronic Data Capture
SMS: Short Message Service
SPIRIT: Standard Protocol Items: Recommendations for Interventional Trials
TA: Technical Assistance
TDF+FTC/3TC: Tenofovir Disoproxil Fumarate with Emtricitabine or Lamivudine
TDF+FTC/3TC+DTG: Tenofovir Disoproxil Fumarate/Lamivudine or Emtricitabine/Dolutegravir
TO: Technical Officer
USD: Unites States Dollar
