# Supplementary material for: Testing different models of pharmacy-based HIV pre- and post-exposure prophylaxis initiation and management in Kenya: protocol for a cluster-randomized controlled trial
Source: Trials. 2025 Dec 30;27:95. doi: 10.1186/s13063-025-09384-7 (PMC12866470; doi:10.1186/s13063-025-09384-7)

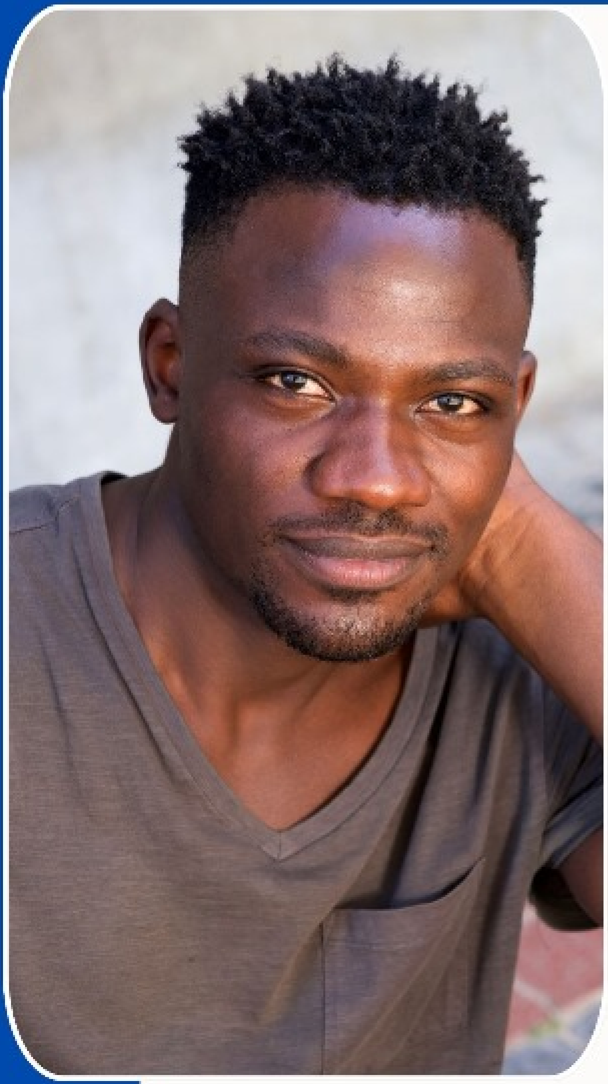

We Stay HIV-Negative with PrEP

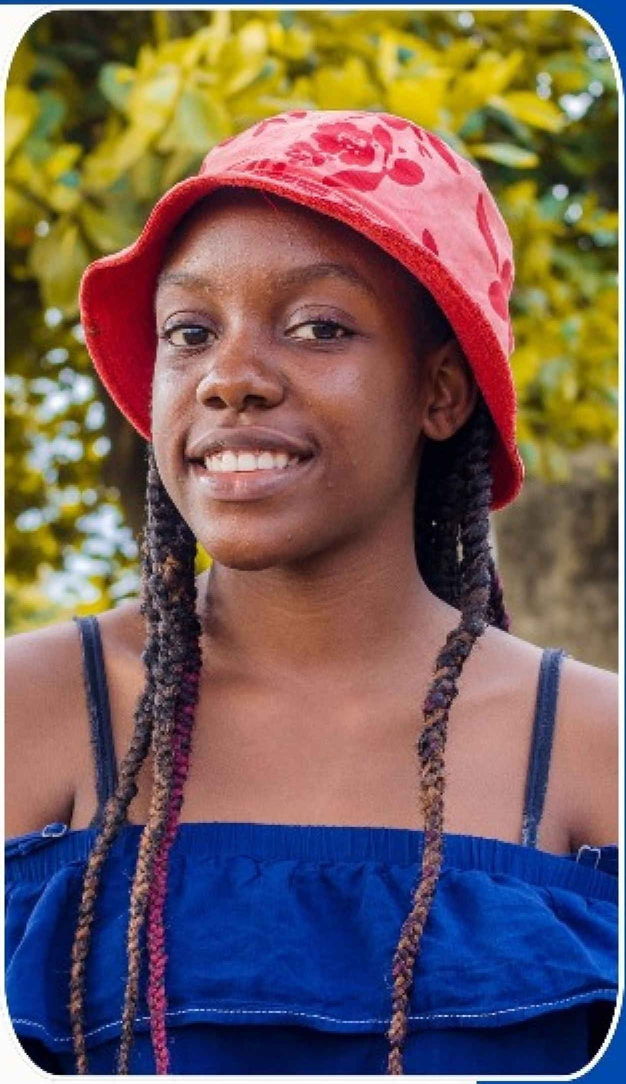

**PrEP is
1-pill-a-day
that keeps us
HIV-negative**

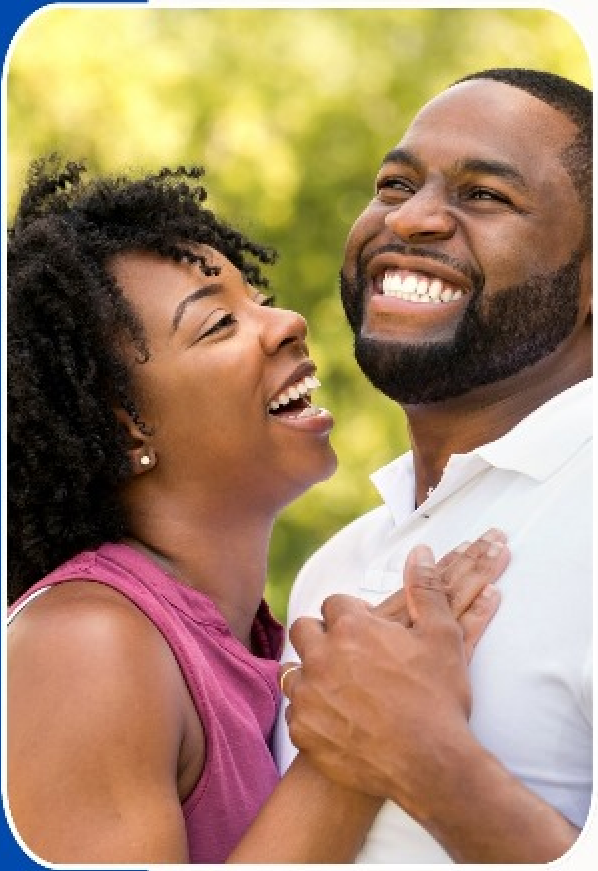

**When we need it
not forever**

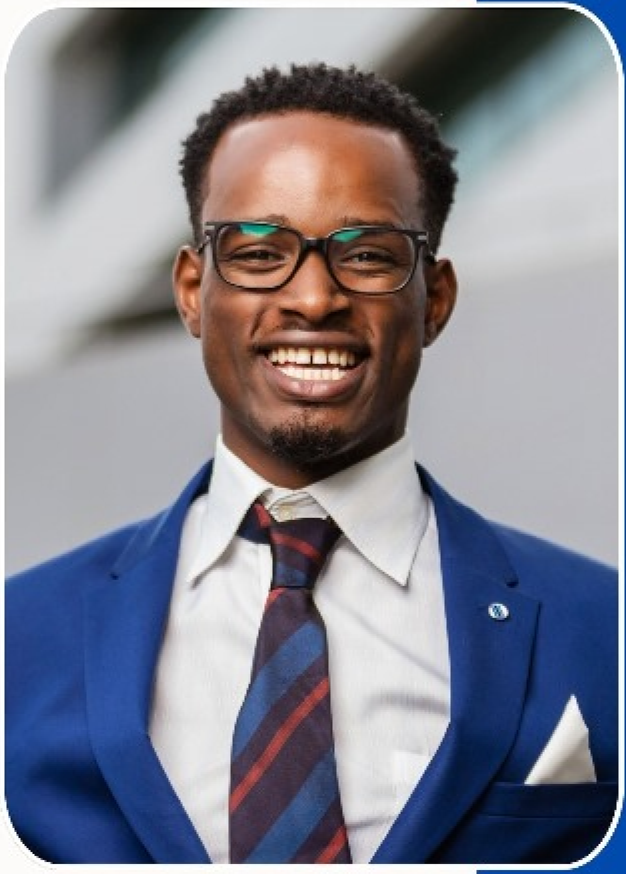

**Ask about PrEP
at the counter**

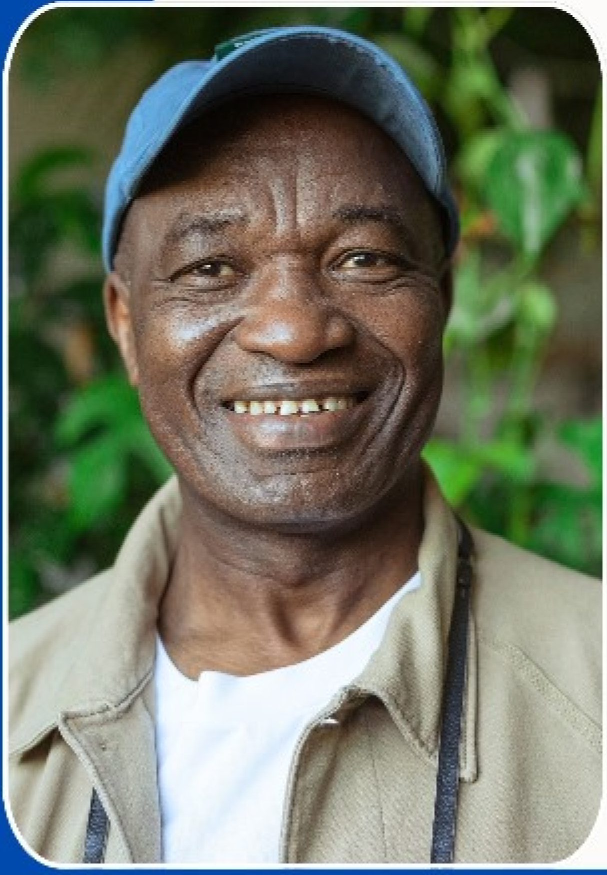

**Call or WhatsApp
0729999999
to learn more**

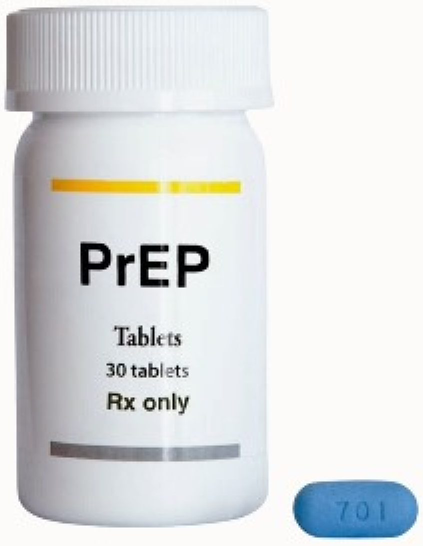

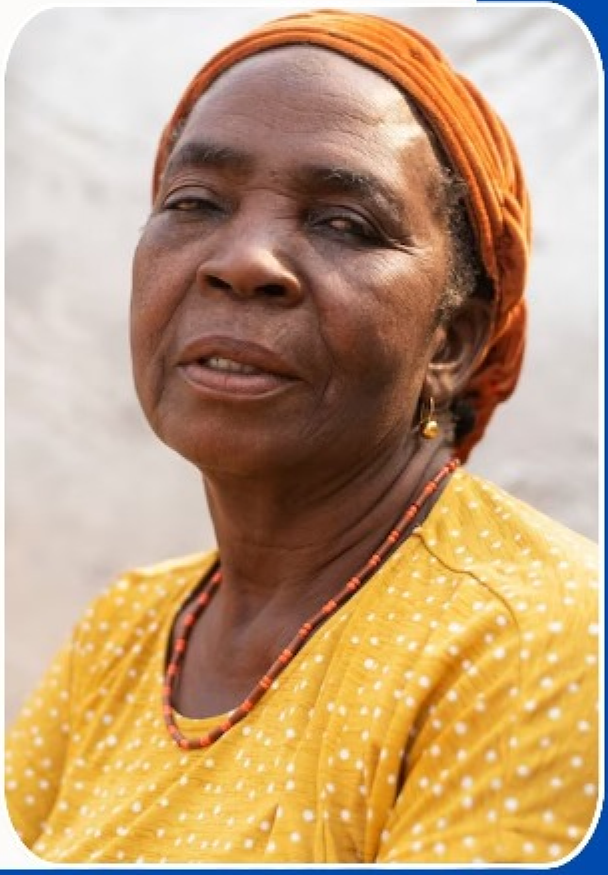

Supplement: Supplementary file 2 — Additional file 2: PrEP poster. This poster was printed out and hung up in all study pharmacies to advertise PrEP services [file 13063_2025_9384_MOESM2_ESM.pdf]
