# Supplementary material for: Testing different models of pharmacy-based HIV pre- and post-exposure prophylaxis initiation and management in Kenya: protocol for a cluster-randomized controlled trial
Source: Trials. 2025 Dec 30;27:95. doi: 10.1186/s13063-025-09384-7 (PMC12866470; doi:10.1186/s13063-025-09384-7)
Supplement: Supplementary file 3 — Additional file 3: PEP poster. This poster was printed out and hung up in all study pharmacies to advertise PEP services. Additional file 4: Fred Hutch IRB approval. Ethical approval document—Fred Hutch IRB. Additional file 5: SERU Kisumu site approval. Ethical approval document—SERU for Kisumu County, Kenya. Additional file 6: SERU Kiambu site approval. Ethical approval document—SERU for Kiambu County, Kenya [file 13063_2025_9384_MOESM3_ESM.pdf]

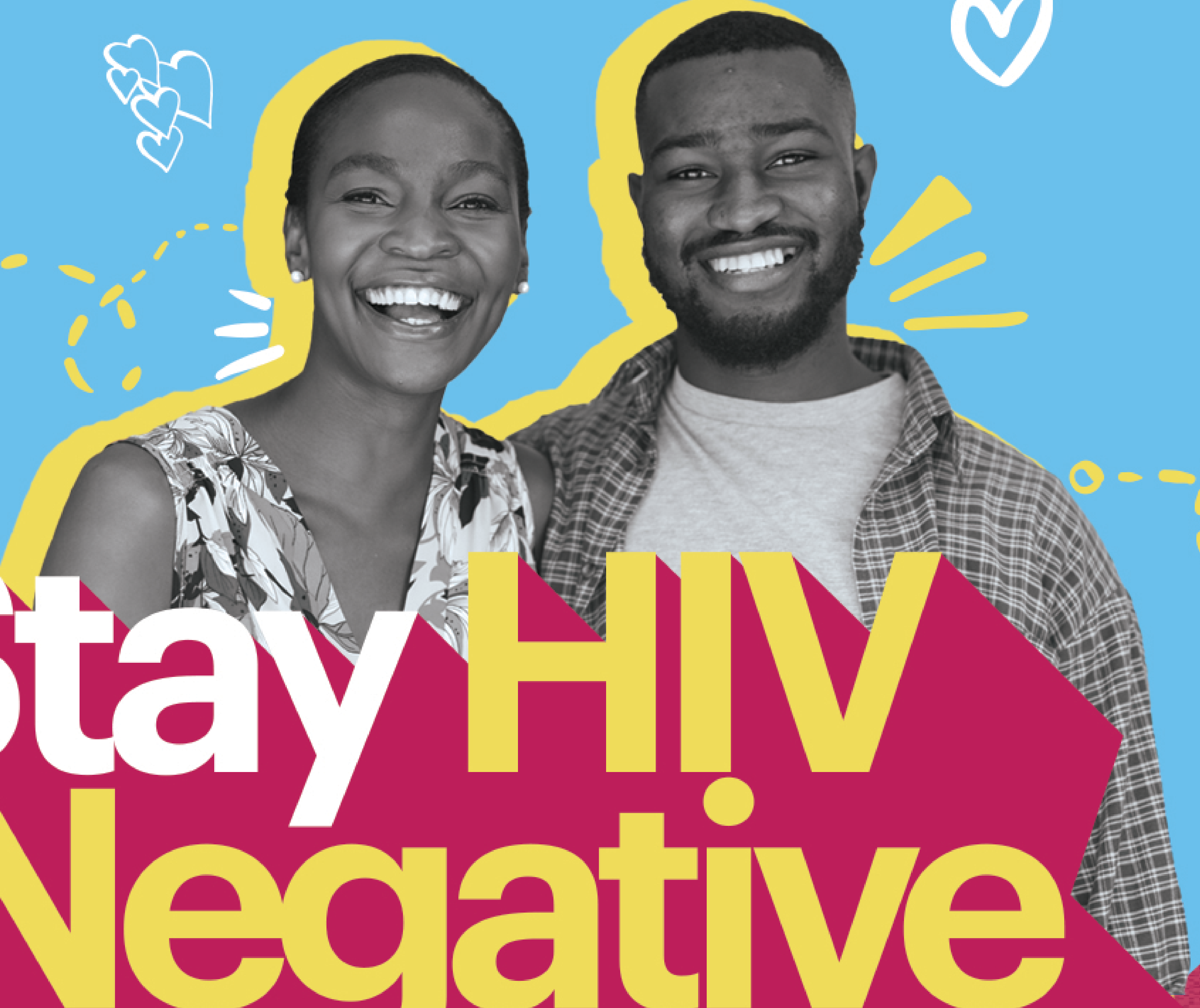

Stay HIV Negative with PEP

PEP is medication that protects us against HIV infection if taken as soon as possible and within 72 hours of a possible exposure.

Ask about PEP at the counter.

Call or WhatsApp
for more information
