# Supplementary material for: Testing different models of pharmacy-based HIV pre- and post-exposure prophylaxis initiation and management in Kenya: protocol for a cluster-randomized controlled trial
Source: Trials. 2025 Dec 30;27:95. doi: 10.1186/s13063-025-09384-7 (PMC12866470; doi:10.1186/s13063-025-09384-7)
Supplement: Supplementary file 4 — Additional file 7: Prescribing Checklist. This prescribing checklist is what providers use to screen clients for PrEP/PEP eligibility. The checklist is uploaded onto a tablet for providers to complete [file 13063_2025_9384_MOESM4_ESM.pdf]

Fred Hutch Cancer Center

Institutional Review Board

APPROVAL – CONTINUING REVIEW

February 15, 2024

Katrina Ortblad
Public Health Sciences Division
Fred Hutchinson Cancer Center

Dear Katrina Ortblad:

On 02/14/2024, the Fred Hutch IRB reviewed and approved the following submission by Full review at Committee A:

Type of Review:	Continuing Review
Study Title:	Pharmacy Delivery to Expand the Reach of PrEP in Kenya: Cluster-Randomized Control Trial
Principal Investigator:	Katrina Ortblad
Submission ID:	CR001-FHIRB0011136
RG Number:	RG1123165
Funding Sources:	BILL AND MELINDA GATES FOUNDATION, N/A, BMGF INV-033052 (Subaward 22-SBA-071)
IND, IDE, or HDE:	None

The IRB approval is effective from 2/14/2024 to 2/13/2025 inclusive. Before 2/13/2025 or within 30 days of study close, whichever is earlier, you are to submit a completed continuing review and required attachments to request continuing approval or closure.

If continuing review approval is not granted before the expiration date of 2/13/2025, approval of this protocol expires on that date.

This IRB re-approval is for research conducted at the following Cancer Consortium site(s):

- Fred Hutch - South Lake Union - Other
- University of Washington

As part of this continuing review, the IRB also reapproved the following participating sites:

- SITE001-FHIRB0011136: JHPIEGO CORPORATION

In conducting this protocol, you are required to follow the requirements listed in the Investigator Manual (HRP-103).

If you have any questions about this submission or the IRB review process, contact Valerie Dossing at vdossing@fredhutch.org or 206-667-5586. The Institutional Review Office (IRO) website at https://extranet.fredhutch.org/en/u/iro.html also contains Policies and Forms used in

the review process. If you have any general questions or suggestions for the IRB, contact the IRO at (206) 667-5900 or iro@fredhutch.org.

Sincerely,

Fred Hutch Institutional Review Board
Committee A: IRB00000021 Committee C: IRB00005619
Committee B: IRB00000022 Committee D: IRB00009831

cc:
Kendall Harkey
Rachel Malen
