# Supplementary material for: Testing different models of pharmacy-based HIV pre- and post-exposure prophylaxis initiation and management in Kenya: protocol for a cluster-randomized controlled trial
Source: Trials. 2025 Dec 30;27:95. doi: 10.1186/s13063-025-09384-7 (PMC12866470; doi:10.1186/s13063-025-09384-7)
Supplement: Supplementary file 6 — Additional file 9: Power analysis for PrEP and PrEP/PEP outcome comparisons (alpha=0.5/3). This table outlines the estimated power calculations by arm and outcome, as well as the number of PrEP and PEP continuations needed by arm to meet >80% power [file 13063_2025_9384_MOESM6_ESM.pdf]

In Search of Better Health

KENYA MEDICAL RESEARCH INSTITUTE

OFFICE OF THE DIRECTOR RESEARCH & DEVELOPMENT

Tell: +254 020 2722541, 2713349, 0722 205 901, 0733 400 003

P.O. Box 54840-00200, Nairobi Email: ddrt@kemri.go.ke
Website: www.kemri.go.ke

KEMRI/RD/22 **May 31, 2023**

TO: **DR. KENNETH NGURE,**
PRINCIPAL INVESTIGATOR,

THROUGH: **DEPUTY DIRECTOR, CCR,**
NAIROBI.

Dear Sir,

RE: PROTOCOL NO. SERU 4649 (*RESUBMISSION III OF INITIAL SUBMISSION*): PHARMACY DELIVERY TO EXPAND THE REACH OF PREP IN KENYA: CLUSTER-RANDOMIZED CONTROL TRIAL (*VERSION 1.2 MARCH 25, 2023*)

Reference is made to your letter dated May 11, 2023. The KEMRI Scientific and Ethics Review Unit (SERU) acknowledges receipt of the revised documents.

This is to inform you that the Committee notes that the issues raised by during the **332nd Committee A** meeting of the KEMRI Scientific Ethics Review Unit (SERU) held on **February 14, 2023** have been adequately addressed.

Consequently, the study is **granted approval** for implementation effective this day, **May 31, 2023** for a period of **one (1) year**. Please note that authorization to conduct this study will automatically expire on **May 30, 2024.** If you plan to continue with data collection or analysis beyond this date, please submit an application for continuation approval to SERU by **April 18, 2024.**

Please note that only approved documents including (informed consents, study instruments, Material Transfer Agreement) will be used. You are required to submit any proposed changes to this study to SERU for review and the changes should not be initiated until written approval from SERU is received. Any unanticipated problems resulting from the implementation of this study should be brought to the attention of SERU and you should advise SERU when the study is completed or discontinued.

Prior to commencing your study, you will be expected to obtain a research license from National Commission for Science, Technology and Innovation (NACOSTI) https://oris.nacosti.go.ke and also obtain other clearances needed.

Yours faithfully,

ENOCK KEBENEI,
THE ACTING HEAD,
KEMRI SCIENTIFIC AND ETHICS REVIEW UNIT.
