# Supplementary material for: Testing different models of pharmacy-based HIV pre- and post-exposure prophylaxis initiation and management in Kenya: protocol for a cluster-randomized controlled trial
Source: Trials. 2025 Dec 30;27:95. doi: 10.1186/s13063-025-09384-7 (PMC12866470; doi:10.1186/s13063-025-09384-7)
Supplement: Supplementary file 7 — Additional file 10: Randomization scheme for the cRCT and back-up pharmacy distribution. This table outlines how many pharmacies were randomized by county and by arm [file 13063_2025_9384_MOESM7_ESM.docx]

**Additional file 7: Prescribing checklists for pharmacy PrEP/PEP: Intervention arms and control arm**

# Prescribing Checklist – *Intervention arms*

| \| Pharmacy: _______________________ Provider name: _______________________________ \| \| --- \| \| **CLIENT PROFILE:**  National or other ID: _____________________________________________ \| \| Name: First ___________________ Middle ___________________ Last ____________________ Telephone no: _________________ \| \| Visit date: *dd / mm / yyyy* Is this client newly enrolling in the study today? ⬜ Yes, newly enrolling ⬜ No, enrolled prior to today \| \| Sex: ⬜ Male ⬜ Female Date of birth: *dd / mm / yyyy* Attends school: ⬜ Yes ⬜ No No. years of school completed: _______  Has ever taken PrEP? ⬜ No ⬜ Yes, current PrEP user ⬜ Yes, former PrEP user 🡪**[If any yes]** Total no. months on PrEP: ______ months  Current marital status: ⬜ Married ⬜ Unmarried 🡪 **[If unmarried, select one]** ⬜ Never married ⬜ Separated/divorced ⬜ Widowed  Relationship status: ⬜ No partners ⬜ Casual partner(s) only ⬜ One primary partner only ⬜ One primary partner and casual partners \| \| What service is this client seeking today? *(Select all that apply)*  ⬜ PrEP initiation ⬜ PrEP follow-up ⬜ PEP initiation ⬜ PEP follow-up \| |
| --- | --- | --- | --- | --- | --- | --- |
| \| **Section 1a: HIV RISK SCREENING** \| \| \| \| --- \| --- \| --- \| \| What is your HIV status? ⬜ Negative ⬜ Unknown ⬜ Positive \| \| ***Stop screening; client is not eligible for PrEP/PEP. Offer to refer to clinic for ART.*** \| \| **Behavioral risk assessment**  Would the client like to self-screen for HIV risk? ⬜ Yes ⬜ No  *Select all that apply in past 6 months, unless noted otherwise.* \| \| ***Pass tablet to client to complete self-screen, then review together.*** \| \| Sex partner(s) is HIV+ AND:  *not on ART, or On ART <6 months, or suspected poor ART adherence, or detectable viral load, or couple trying to conceive*  Sex partner(s) high risk & HIV status is unknown:  Has sex with >1 partner  Ongoing intimate partner/gender-based violence  Transactional sex  Recent STI  Recurrent use of post-exposure prophylaxis (PEP)  Recurrent sex under influence of alcohol/recreational drugs  Inconsistent or no condom use  Injection drug use with shared needles and/or syringes \| ⬜ Yes ⬜ No  ⬜ Yes ⬜ No  ⬜ Yes ⬜ No  ⬜ Yes ⬜ No  ⬜ Yes ⬜ No  ⬜ Yes ⬜ No  ⬜ Yes ⬜ No  ⬜ Yes ⬜ No  ⬜ Yes ⬜ No \|  \| \| Do you think you’re at risk of getting HIV? \| ⬜ Yes ⬜ No \|  \| \| Thinking about your behaviors in the next month, do you those behaviors put you at low, medium, or high risk of getting HIV?  ⬜ Low ⬜ Medium ⬜ High ⬜ Don’t know \| \| \| \| Population type: ⬜Gen Pop ⬜ Discordant couple ⬜Key Population 🡪**[Specify:]** ⬜MSM ⬜MSW ⬜ FSW ⬜ PWID ⬜Prefer not to answer \| \| \| |
| \| **Section 1b: PEP SCREENING** \| \| \| \| --- \| --- \| --- \| \| **In the past 72 hours, did you experience any of the following with someone who may be living with HIV or whose HIV status you don’t know?** \| \|  \| \| Condom break or condomless sex \| ⬜Yes ⬜ No \|  \| \| Shared needles/syringes to inject drugs or accidental needle stick (e.g., healthcare worker) \| ⬜Yes ⬜ No \|  \| \| Sexual assault \| ⬜Yes ⬜ No \|  \| \| Other high-risk type & material exposure *(specify)*: __________ ___________________________________________________ \| ⬜Yes ⬜ No \|  \| \| **Section 1c. SERVICE CANDIDACY DETERMINATION** \| \| \| \| **After talking with this client, which service might benefit them the most?** *(Select one)*  ⬜PrEP initiation ⬜ PrEP continuation ⬜ PEP initiation ⬜ Neither PrEP nor PEP 🡪 **[If neither:]** Reason: ⬜ No HIV risk ⬜ Not interested/ready ⬜Other \| \| \| \| **Section 2. MEDICAL SAFETY ASSESSMENT *Display only if 1c = “PrEP” or “PEP”*** \| \| \| \| **[For PrEP/PEP follow-up:]** Severe side effects from PrEP/PEP use: *(Select all that apply)* \| \| ***Stop screening.***  ***Contact remote clinician, refer to clinic, and complete Referral Form below.*** \| \| ⬜ Headache ⬜ Nausea ⬜ Abdominal discomfort  ⬜ Weight loss ⬜ Vomiting ⬜ Fatigue  ⬜ Diarrhea ⬜ Bloating ⬜ Flatulence  ⬜ Other *(specify):* _______________________________ \| ⬜ None \| ***Continue screening, but offer to refer to clinic at end and consult remote clinician as needed.*** \| \| **[For PrEP initiation:]** Any history of: *(Select all that apply)* \|  \| \| ⬜ Kidney disease ⬜ Liver disease ⬜ Diabetes ⬜ Hypertension \|  \| \| **[For PrEP initiation]:** Is client currently taking any medications? \| ⬜ Yes ⬜ No \| \| **[If yes]** Are any of these medications diuretics? \| ⬜ Yes ⬜ No \| \| **[For PrEP/PEP initiation:]**  Any symptoms of acute HIV infection? *(Select all that apply)*   \| ⬜ Ulcers (sores) in mouth, esophagus, anus, or genitals \| \| \| \| --- \| --- \| --- \| \| ⬜ Headache \| ⬜ Fatigue \| ⬜ Aching muscles \| \| ⬜ Sore throat \| ⬜ Fever \| ⬜ Swollen lymph nodes \| \| ⬜ Rash that does not itch (usually on torso) \| \| \| \| Only if 4 or more  ⬜ None \| \| **[For STIs:]** In the last 7 days, any of the following symptoms? *(Select all that apply)* \| \| \| ⬜ Vaginal/urethral discharge or itching ⬜ Rectal pain  ⬜ Pain or burning while urinating ⬜ Painful vaginal sex  ⬜ Pelvic/low abdominal pain ⬜ Sores on genital area \| ⬜ None \|  \| |
| \|  \| \| \| \| --- \| --- \| --- \| \| **Section 3. INFORMED CONSENT – *Displays only if client passes medical safety assessment & only for initiation visits.*** \| \| \| \| The information you’ve shared with me indicates that you might benefit from taking [PrEP/PEP] to prevent HIV. As part of a research study, this pharmacy is offering [PrEP/PEP] services to clients like you, so long as they test negative for HIV. If you are interested, I could call a research assistant who will provide you with more information about the study, ask you some questions to make sure you’re eligible for the study, and enroll you. Would you like to talk to the research assistant to hear more about the study?  ⬜ Yes ⬜ No \| \| \| \| **[If no]** Why aren’t you interested in hearing more about this study? *(Select all that apply)*   \| ⬜In a rush today/not enough time \| ⬜Don’t want to get tested for HIV at pharmacy \| ⬜Need more time to think it over \| \| --- \| --- \| --- \| \| ⬜Don’t want to participate in research \| ⬜**[Arm 1 pharmacies only]** I don’t want to pay for PrEP/PEP \| ⬜Other \| \| \| \| \| ***[If “Yes” selected above, verbal consent script specific to pharmacy’s study arm & region displays here]***  ***[Question for client to complete:]*** Do you consent to participate in this study? ⬜ I AGREE ⬜ I DO NOT AGREE \| \| \| \| **Section 4. COUNSELING – *Display only if client selected “I AGREE” in Section 3*** \| \| \| \| Adherence counseling done (if applicable):  PrEP/PEP side effect counseling done (if applicable): \| ⬜ Yes ⬜ No ⬜ N/A  ⬜ Yes ⬜ No ⬜ N/A \| ***Warning: Please complete counseling (if applicable) before proceeding to HIV testing.*** \| \| **Section 5. HIV TESTING – *Display only if client selected “I AGREE” in Section 3***  ***DO NOT give PrEP or PEP.***  ***Client not eligible unless undergoes HIV testing and tests negative on a Determine test. Offer to refer to clinic.*** \| \| \| \| Was the client tested for HIV today? ⬜ Yes ⬜ No  **[If yes]** Determine test result ⬜ Negative ⬜ Positive ⬜ Invalid \| \| ***Retest* the client using a First Response test kit.** \| \| ***Upload here***  Upload photo of Determine test here:  **[If invalid:]** 2^nd^ (repeat) Determine test result: ⬜ Negative ⬜ Positive ⬜ Invalid  ***Upload here***  Upload photo of 2^nd^ (repeat) Determine test here:  **[If Determine test result is Positive or 2^nd^ (repeat) Determine test result is Positive]**:  ***Regardless of First Response result, refer client to clinic for confirmatory testing and complete Referral Form.***  First Response test result: ⬜ Negative ⬜ Positive ⬜ Invalid  ***Upload here***  Upload photo of First Response test here: \| \| ***Retest* the client using a new Determine test kit.**  ***Retest* the client using a First Response test kit.**  ***Refer client to clinic for confirmatory testing, complete Referral Form, & contact remote clinician.*** \| \| **Section 6. DISPENSING & TCA *Display only if client selected “I AGREE” in Section 3*** \| \| \| \| **Client was dispensed today:** *(Select one)*  ⬜ Nothing  ⬜ PEP: TDF+3TC+DTG  ⬜ PrEP 🡪 **[If PrEP, select regimen:]** ⬜ TDF-FTC ⬜ TDF-3TC \| \| \| \| **[If nothing dispensed:]** Reason why nothing dispensed: *(check all that apply)*  ⬜Positive HIV test ⬜ Not ready to start PrEP/PEP ⬜ Concerned about side effects ⬜ Wants to consult partner  ⬜ Wants to HIV test at home ⬜ Other: ________________________________________________________  **# PrEP/PEP pills dispensed today: _________ pills** ⬜ N/A **Next appointment date:** *dd / mm / yyyy* \| \| \| \| **Section 7. REFERRAL** \| \| \| \| **Upload photo of referral form:**  ***Upload photo of Referral Form here*** \| \| \| \| **Section 8: NOTES** \| \| \| \| **OPTIONAL: Pharmacy provider comments/observations: _______________________________________________________________**  **________________________________________________________________________________________________________________** \| \| \| |
|  |

# Prescribing Checklist – *Referral (control) arm*

| \| Pharmacy: _______________________ Provider name: _______________________________ \| \| --- \| \| **CLIENT PROFILE:**  National or other ID number: _________________________________________ \| \| Name: First ___________________ Middle ___________________ Last ____________________ Telephone no: _________________ \| \| Visit date: *dd / mm / yyyy* \| \| Sex: ⬜ Male ⬜ Female Date of birth: *dd / mm / yyyy* Attends school: ⬜ Yes ⬜ No No. years of school completed: _______  Has ever taken PrEP? ⬜ No ⬜ Yes, current PrEP user ⬜ Yes, former PrEP user 🡪 **[If any yes]** total no. months on PrEP: ______ months  Current marital status: ⬜ Married ⬜ Unmarried 🡪 **[If unmarried, select one]** ⬜ Never married ⬜ Separated/divorced ⬜ Widowed  Relationship status: ⬜ No partners ⬜ Casual partner(s) only ⬜ One primary partner only ⬜ One primary partner and casual partners \| \| What service is this client seeking today? *(Select all that apply)*  ⬜ PrEP screening ⬜ PEP screening \| |
| --- | --- | --- | --- | --- | --- | --- |
| \| **Section 1a: HIV RISK SCREENING** \| \| \| \| --- \| --- \| --- \| \| What is your HIV status? ⬜ Negative ⬜ Unknown ⬜ Positive \| \| ***Stop screening; client is not eligible for PrEP/PEP. Offer to refer to clinic for ART.*** \| \| **Behavioral risk assessment**  Would the client like to self-screen for HIV risk? ⬜ Yes ⬜ No  *Select all that apply in past 6 months, unless noted otherwise.* \| \|  \| \| Sex partner(s) is HIV+ AND:  *not on ART, or On ART <6 months, or suspected poor ART adherence, or detectable viral load, or couple trying to conceive*  Sex partner(s) high risk & HIV status is unknown:  Has sex with >1 partner  Ongoing intimate partner/gender-based violence  Transactional sex  Recent STI  Recurrent use of post-exposure prophylaxis (PEP)  Recurrent sex under influence of alcohol/recreational drugs  Inconsistent or no condom use  Injection drug use with shared needles and/or syringes \| ⬜ Yes ⬜ No  ⬜ Yes ⬜ No  ⬜ Yes ⬜ No  ⬜ Yes ⬜ No  ⬜ Yes ⬜ No  ⬜ Yes ⬜ No  ⬜ Yes ⬜ No  ⬜ Yes ⬜ No  ⬜ Yes ⬜ No \| ***Pass tablet to client to complete self-screen, then review together.*** \| \| Do you think you’re at risk of getting HIV? \| ⬜ Yes ⬜ No \|  \| \| Thinking about your behaviors in the next month, do you think those behaviors would put you at low, medium, or high risk of getting HIV?  ⬜ Low ⬜ Medium ⬜ High ⬜ Don’t know \| \| \| \| Population type: ⬜Gen Pop ⬜ Discordant couple ⬜Key Population🡪 **[Specify:]** ⬜MSM ⬜MSW ⬜ FSW ⬜ PWID ⬜Prefer not to answer \| \| \| |
| \| **Section 1b: PEP SCREENING** \| \| \| \| \| \| --- \| --- \| --- \| --- \| --- \| \| **In the past 72 hours, did you experience any of the following with someone who may be living with HIV or whose HIV status you don’t know?** \| \| \| \| \| \| Condom break or condomless sex \| \| ⬜Yes ⬜ No \| \|  \| \| Shared needles/syringes, to inject drugs or accidental needle stick (e.g., healthcare worker) \| \| ⬜Yes ⬜ No \| \|  \| \| Sexual assault \| \| ⬜Yes ⬜ No \| \|  \| \| Other high-risk type & material exposure *(specify)*: ____________  ___________________________________________________ \| \| ⬜Yes ⬜ No \| \|  \| \| **Section 1c: SERVICE CANDIDACY DETERMINATION** \| \| \| \| \| \| **After talking with this client, which service might benefit them the most?** *(select one)*  ⬜ PrEP ⬜ PEP ⬜ Neither PrEP nor PEP 🡪 **[If neither]** Reason: ⬜ No HIV risk ⬜ Not interested/ready \| \| \| \| \| \| **Section 2. MEDICAL SAFETY ASSESSMENT *Display only if 1c = “PrEP” or “PEP”*** \| \| \| \| \| \| **[For PrEP/PEP screening:]** Any symptoms of acute HIV infection? *(Select all that apply)* \| \|  \| \|  \| \| ⬜ Ulcers (sores) in mouth, esophagus, anus, or genitals \| \| ⬜ None  Only if 4 or more \| \|  \| \| ⬜ Headache  ⬜ Sore throat \| ⬜ Fatigue ⬜ Aching muscles  ⬜ Fever ⬜ Swollen lymph nodes \| \| ⬜ Rash that does not itch (usually on torso) \| \| ***Stop screening.***  ***Refer to clinic, and complete Referral Form below.*** \| \| **[For PrEP screening:]** Is client currently taking any medications?  **[If yes]** Are any of these medications diuretics? \| \| ⬜Yes ⬜ No  ⬜Yes ⬜ No \| \|  \| \| **[For PrEP screening:]** Any history of: *(select all that apply)*  ⬜ Kidney disease ⬜Liver disease ⬜ Diabetes ⬜ Hypertension \| \| \|  \|  \| \| **[For STIs:]** In the last 7 days, any of the following symptoms? *(Select all that apply)* \| \| \| \|  \| \| ⬜ Vaginal/urethral discharge or itching ⬜ Rectal pain  ⬜ Pain or burning while urinating ⬜ Painful vaginal sex  ⬜ Pelvic/low abdominal pain ⬜ Sores on your genital area \| \| \| ⬜ None \| ***Continue screening, but offer to refer to clinic at end.*** \| |
| \| **Section 3. INFORMED CONSENT *Displays only if client passes medical safety assessment.*** \| \| \| --- \| --- \| \| The information you’ve shared with me indicates that you might benefit from taking [PrEP/PEP] to prevent HIV. As part of a research study, this pharmacy is offering clients like you a free referral to a nearby clinic for [PrEP/PEP] services. If you are interested, I can call a research assistant who will provide you with more information about the study, ask you some questions to make sure you’re eligible for the study, and enroll you. Would you like to talk to the research assistant to hear more about the study?  ⬜ Yes ⬜ No \| \| \| **[If no]** Why aren’t you interested in hearing more about this study? *(Select all that apply)*   \| ⬜In a rush today/not enough time \| ⬜Need more time to think it over \| ⬜Other \| \| --- \| --- \| --- \| \| ⬜Don’t want to participate in research \| ⬜ I don’t want to to get PrEP/PEP at a clinic \|  \| \| \| \| ***[If “Yes” selected above, verbal consent script specific to pharmacy’s region displays here]***  *[****Question for client to complete:]*** Do you consent to particpate in this study? ⬜ I AGREE ⬜ I DO NOT AGREE \| \| \| **Section 4. HIV TESTING *Display only if client selected “I AGREE” in Section 3*** \| \| \| Did the client purchase an HIV test kit or get tested for HIV today? ⬜Yes ⬜ No  **[If yes]** Type of HIV test: ⬜Oral-fluid self-test ⬜ Blood-based self-test ⬜ Other  Brand of HIV test: *_____________________________* Amount charged? *KES:* *_______*  Did you help the client with testing? ⬜Yes ⬜ No  HIV test result ⬜ Don’t know ⬜ Negative ⬜ Positive ⬜ Invalid  Upload photo of HIV test here:  ***Upload here*** \| ***Refer to clinic for confirmatory testing/ART services and complete Referral Form.*** \| \| **Section 5. REFERRAL SLIP** \| \| \| **Did the client accept to be referred?** ⬜ Yes ⬜ No  ***[If yes]* Upload photo of referral form:**  ***Upload photo of referral form here*** \| \| \| **[If no] Why not?** *(Select all that apply)* ⬜Too busy ⬜Don’t like going to clinics ⬜Don’t feel at HIV risk  ⬜Unsure/need more time ⬜Other: ________________________________ \| \| \| **Section 6: NOTES** \| \| \| **OPTIONAL: Pharmacy provider comments/observations: _______________________________________________________________**  **________________________________________________________________________________________________________________** \| \| |

***Refer to clinic for confirmatory testing/ART services and complete Referral Form.***
