# Supplementary material for: Testing different models of pharmacy-based HIV pre- and post-exposure prophylaxis initiation and management in Kenya: protocol for a cluster-randomized controlled trial
Source: Trials. 2025 Dec 30;27:95. doi: 10.1186/s13063-025-09384-7 (PMC12866470; doi:10.1186/s13063-025-09384-7)
Supplement: Supplementary file 8 — Additional file 11. Qualitative interviews_2025.11.05. This describes the methodological orientation for the qualitative research of this project, as well as the rationale for the type and number of in-depth interviews collected. Additional file 12: Original funding document. BMGF INV-033052 investment document. The original funding document for this grant [file 13063_2025_9384_MOESM8_ESM.docx]

**Additional File 8. Number of PrEP and PEP initiations and continuations expected per pharmacy per month, by study arm^a^**

| **Outcome** | **Arm 1:**  **Client-sustained** | **Arm 2: Implementor-sustained** | **Arm 3:**  **HTS-supported** | **Arm 4:**  **Referral (SOC)** |
| --- | --- | --- | --- | --- |
| **PrEP initiations*** | 8 | 11 | 13 | 5 |
| **PrEP continuations*** | 4 | 7.7 | 10.4 | 3 |
| PEP initiations | 2 | 3 | 4 | 1 |
| PEP continuations | 0.3 | 0.6 | 0.9 | 0.2 |
| PrEP/PEP initiations | 10 | 14 | 17 | 6 |
| PrEP/PEP continuations | 4.3 | 8.3 | 11.3 | 3.2 |
| Abbreviations: HIV testing services counselor (HTS); post-exposure prophylaxis (PEP); pre-exposure prophylaxis (PrEP); standard of care (SOC)  ^a^ Assumptions based on pilot data; an implementation period of 16 months assumed.  *Co-primary outcomes | | | | |
