# Supplementary material for: Testing different models of pharmacy-based HIV pre- and post-exposure prophylaxis initiation and management in Kenya: protocol for a cluster-randomized controlled trial
Source: Trials. 2025 Dec 30;27:95. doi: 10.1186/s13063-025-09384-7 (PMC12866470; doi:10.1186/s13063-025-09384-7)
Supplement: Supplementary file 5 — Additional file 8: Initiation assumptions. Number of PrEP and PEP initiations and continuations expected per pharmacy per month, by study arm. This table outlines the number of PrEP, PEP, and PrEP/PEP initiations and continuations we anticipated per pharmacy per month [file 13063_2025_9384_MOESM5_ESM.pdf]

KENYA MEDICAL RESEARCH INSTITUTE

P.O. Box 54840-00200, NAIROBI, Kenya
Tel: (254) 2722541, 2713349, 0722-205901, 0733-400003, Fax: (254) (020) 2720030
Email: director@kemri.org, info@kemri.org, Website. www.kemri.org

Final Decision Certificate

This document certifies that the study:

"Pharm PrEP: cRCT"

Principal Investigator: Professor Bukusi, Elizabeth

Reference number: SERU4645/

Was reviewed and received the following status:

"done"

Additional Comments: Final decision: **approved**
Comments sent:
This is to Inform you that during the 331st Committee B meeting of the KEMRI Scientific and Ethics Review Unit (SERU) held on January 18, 2023, the above study was discussed.

The Committee received and reviewed the following documents:
1. Study protocol version 1.1 dated December 19, 2022
2. Study information sheets version 1.0 dated December 16, 2022 in English, Dholuo and Kiswahili
3. Client verbal consent scripts version 1.0 dated December 16, 2022 in English, Dholuo and Kiswahili
4. Informed consent for clients opting to provide an optional DBS sample version 1.1 dated December 16, 2022 in English Dholuo and Kiswahili
5. Informed consent form for providers version 1.1 dated December 16, 2022

2022 in English, Dholuo and Kiswahili

12. Pharmacy PrEP cRCT: pharmacy provider interview guide

13. Pharmacy PrEP cRCT: remote clinician interview guide

14. Pharmacy PrEP cRCT: client exit survey

15. PrEP brochure

16. PrEP poster

17. Point by point response to CMR CSC reviewers' comments dated 15th November 2022

18. Translation and back translation certificates for all translated documents

19. CV of all non-KEMRI investigators

20. Investigators ethics certificates

21. Proof of payment

The Committee noted that the above referenced study aims to test pharmacy-based models as an alternative venue for PrEP/PEP delivery.
Consequently, the study is granted approval for implementation effective this day, January 18, 2023 for a period of one year. Please note that authorization to conduct this study will automatically expire on January 17, 2024. If you plan to continue with data collection or analysis beyond this date, please submit an application for continuation approval to SERU by December 7, 2023.

Please note that only approved documents including (informed consents, study instruments, Material Transfer Agreement) will be used. You are required to submit any proposed changes to this study to SERU for review and the changes should not be initiated until written approval from SERU is received. Any unanticipated problems resulting from the implementation of this study should be brought to the attention of SERU and you should advise SERU when the study is completed or discontinued.

Prior to commencing your study, you will be expected to obtain a research license from National Commission for Science, Technology and Innovation (NACOSTI) https://oris.nacosti.go.ke and also obtain other clearances needed.

Yours faithfully,

ENOCK KEBENEI,
THE ACTING HEAD,
KEMRI SCIENTIFIC AND ETHICS REVIEW UNIT.
